# One-Pot Synthesis of Strong Anionic/Charge-Neutral Amphiphilic Block Copolymers

**DOI:** 10.1021/acsmacrolett.3c00355

**Published:** 2023-07-18

**Authors:** Théophile Pelras, András Eisenga, Gábor Érsek, Aldo Altomare, Giuseppe Portale, Marleen Kamperman, Katja Loos

**Affiliations:** † Macromolecular Chemistry and New Polymeric Materials, Zernike Institute for Advanced Materials, University of Groningen, Nijenborgh 4, 9747 AG Groningen, The Netherlands; ‡ Polymer Science, Zernike Institute for Advanced Materials, University of Groningen, Nijenborgh 4, 9747 AG Groningen, The Netherlands; § Physical Chemistry of Polymeric and Nanostructured Materials, Zernike Institute for Advanced Materials, University of Groningen, Nijenborgh 4, 9747 AG Groningen, The Netherlands

## Abstract

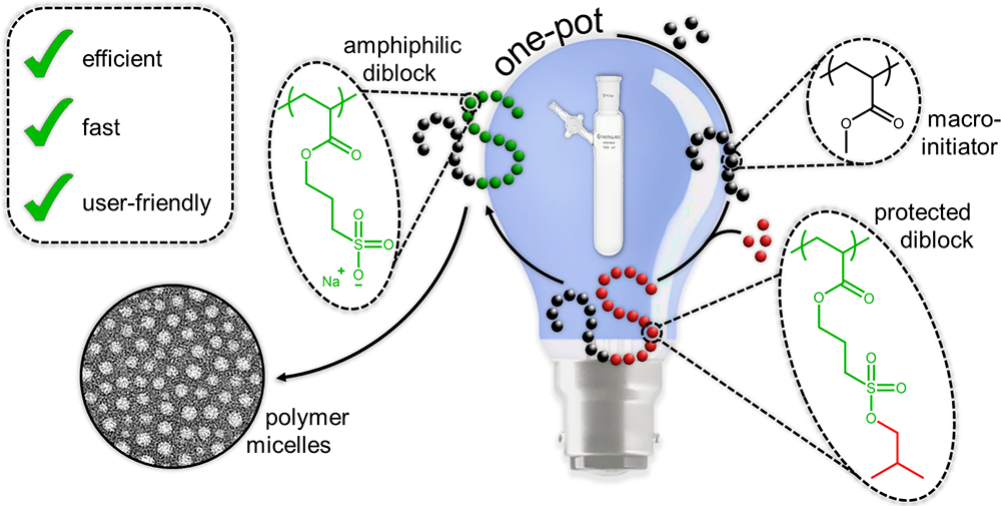

Despite the ever more versatile polymerization techniques that are becoming available, the synthesis of macromolecules with tailored functionalities can remain a lengthy endeavor. This becomes more conspicuous when the implementation of incompatible chemistries (i.e., strong polyelectrolytes) within sequence-controlled polymers is desired, often requiring (i) polymerization, (ii) chain extension, and (iii) postpolymerization modification. Herein, we explore the production of strong anionic/charge-neutral block copolymers (BCPs) in a one-pot fashion. This straightforward three-step process includes the synthesis of a macroinitiator and chain extension via rapid and efficient photomediated atom transfer radical polymerization, followed by in situ deprotection to expose the polyanionic domains. The resulting BCPs, which are strong amphiphiles by nature, are capable of self-assembly in aqueous media, as evidenced by dynamic light scattering, small-angle X-ray scattering, ζ-potential measurements, and transmission electron microscopy. We further demonstrate the versatility of our methodology by producing several BCPs through sampling of a single reaction mixture, enabling the straightforward production of strong polymer amphiphiles.

The fields of macromolecular chemistry and engineering strive to produce materials of increasing complexity, featuring dynamic behaviors and/or capable of self-assembly into ordered structures.^1^ However, the synthesis of sequence-controlled macromolecules possessing unusual architectures (e.g., bottlebrushes)^2,3^ or chemistry (e.g., strong polyelectrolytes)^4,5^ remains challenging and time-consuming and often involves several reaction steps with intermediate purification and characterization. A strategy proposed to circumvent this issue lies in one-pot reactions, i.e., complex multistep syntheses performed in a single vessel with no intermediate purification. Such processes would enable streamlined polymerization steps, deprotection, and/or attachment of functional groups and offer significant reduction of the number of catalysts and solvents needed as well as far shorter production times.

However, this promising strategy has seen limited outcomes for the production of tailored block copolymers (BCPs), mainly because reactions need to be performed either orthogonally or sequentially with the same catalytic system. A handful of elegant examples have nonetheless been reported, such as the production of glycosylated nanoparticles through a combination of thiolactone opening, nucleophilic substitution, and deprotection.^6^ Alternatively, BCPs can be produced in a one-pot process through a combination of orthogonal polymerization techniques, such as ring-opening polymerization (ROP) and reversible addition–fragmentation chain-transfer (RAFT) polymerization from a bis-functional chain-transfer agent^7^ or the installation of a chain-transfer agent onto living cationic chain, followed by RAFT polymerization.^8^ One-pot processes have also been proposed for the more straightforward synthesis of complex bottlebrush architectures, notably through sequential ROPs to produce bottlebrushes^9^ and comblike polymers^10^ or through a combination of ROP and RAFT.^11^ Some reactions, such as atom-transfer radical polymerization (ATRP) and azide–alkyne Huisgen cycloaddition, can be conducted with the same catalyst, which enables sequential one-pot polymerization/coupling^12^ or even a simultaneous process.^13^

However, to the best of our knowledge, no one-pot system has yet featured the modification of polymer chains to include permanent charges. Strong polyanions (i.e., macromolecules whose charge density is independent of the medium’s pH) are notoriously challenging to produce efficiently, all the more so within BCPs featuring hydrophobic segments (i.e., charge-neutral/anionic block copolymers). The direct insertion of an anionic block requires the use of stabilizing agents,^14,15^ solvent mixtures,^16−18^ or high copper concentrations when ATRP techniques are involved,^18^ all of which hamper in-depth characterization of the final product. The later installation of charges, e.g., sulfonation of polystyrene, often requires harsh conditions^19^ and leads to many side reactions,^20^ while protected monomers exhibit low reactivity and deprotection yields.^21,22^ However, such systems offer great potential for the engineering of active surfaces^23,24^ or for the fabrication of proton-exchange membranes;^25^ thus, a straightforward methodology capable of producing such macromolecules remains of high interest.

We have recently reported a robust route for the synthesis of BCPs featuring both hydrophobic and sulfopropyl-based hydrophilic segments through reversible deactivation radical polymerization (RDRP) techniques^26,27^ and have further demonstrated the feasibility of the *in situ* modification of an isobutoxy-protected homopolymer,^28^ paving the way for a more straightforward production of BCPs featuring strong polyelectrolyte blocks. Herein, we present a methodology for the facile and efficient synthesis of BCPs possessing both hydrophobic and strong anionic segments (i.e., including polymerization of a first block, chain extension, and postpolymerization modification, Scheme 1) in a one-pot fashion using fast and user-friendly photo-ATRP.^29^

**Scheme 1:**
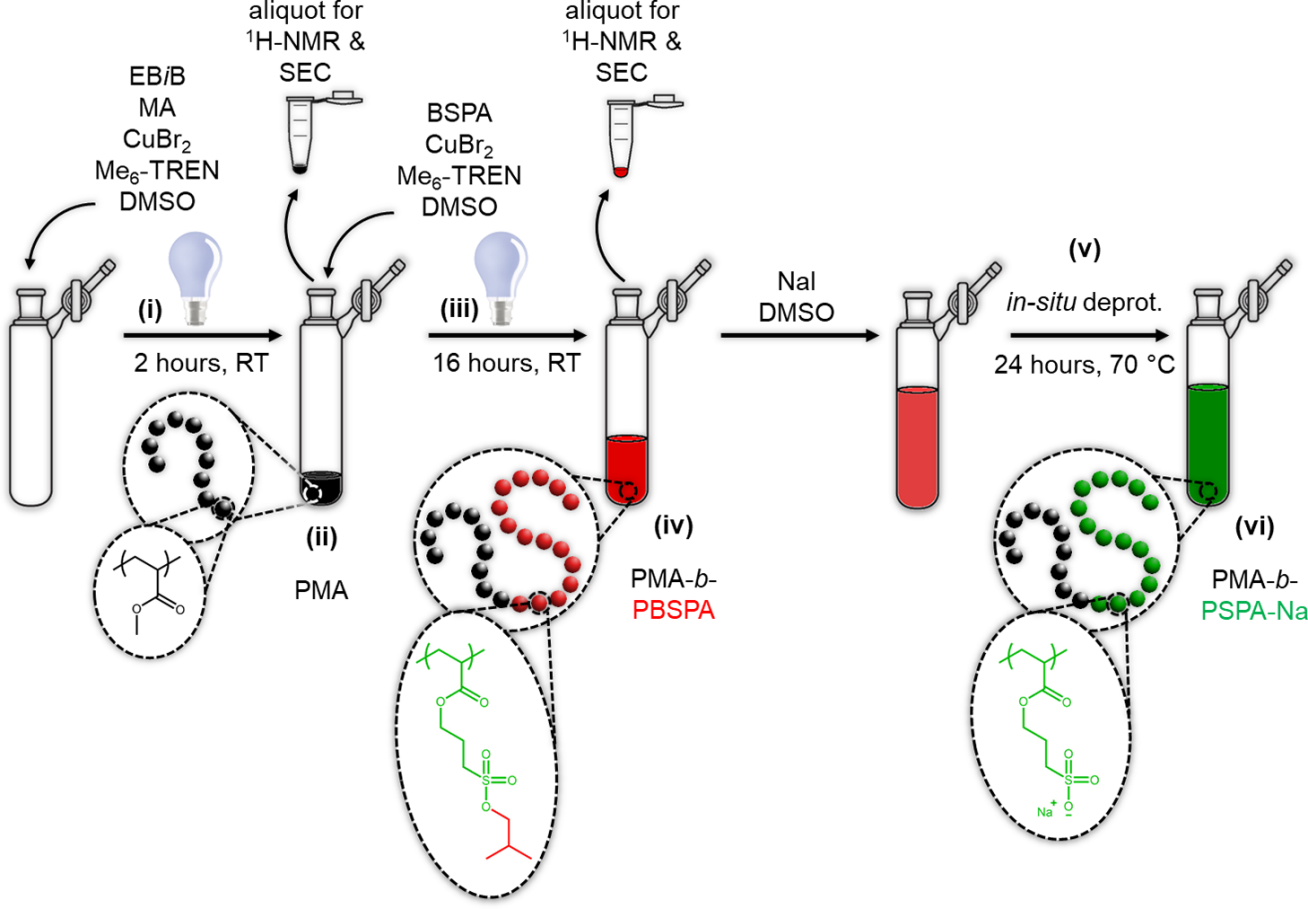
One-Pot Synthesis of Strong Amphiphiles Featuring a Polyanionic Segment (i) A first photo-ATRP step enables the formation of (ii) a poly(methyl acrylate) macroinitiator before (iii) *in situ* chain extension with 3-isobutoxysulfopropyl acrylate to yield (iv) a protected poly(methyl acrylate)-*block*-poly(3-isobutoxysulfopropyl acrylate) copolymer; a weak nucleophile and solvent are added into the same vessel before (v) *in situ* deprotection, yielding (vi) a poly(methyl acrylate)-*block*-poly(sulfopropyl acrylate sodium salt) amphiphilic block copolymer.

Amidst the large variety of RDRP techniques, we selected UV-catalyzed copper-mediated polymerization^30−32^ due to its ease of setup as well as rapid kinetics, low working temperatures, and amounts of catalyst, while preserving high end-group fidelity and low dispersities (Figure S1). First, we tested the photopolymerization of our protected monomer using ethyl α-bromoisobutyrate (EB*i*B, 1 equiv) as the initiator, tris[2-(dimethylamino)ethyl]amine (Me_6_-TREN, 0.12 equiv) as the ligand, copper(II) bromide (CuBr_2_, 0.02 equiv) as a copper source/deactivator, and dimethyl sulfoxide (DMSO) as the solvent. Almost full conversion was achieved within 3 h at room temperature, while maintaining low dispersity and avoiding side reactions, as evidenced by proton nuclear magnetic resonance (^1^H NMR) spectroscopy and size exclusion chromatography (SEC). After purification, a poly(3-isobutoxysulfopropyl acrylate) homopolymer (PBSPA_99_, *M*_n,NMR_ = 24900 Da, *Đ* = 1.12, Figure S2-1) was obtained, with excellent correlation between the signals of the isobutoxy group and the alkyl spacer, indicating that no unwanted deprotection occurred. Removal of the protective groups can easily and efficiently be performed in dimethyl sulfoxide using 3 equiv of NaI (deprotection yield ≥ 99%,Figure S3) to yield a strong polyanion.^27^ With a similar conversion value reached for a poly(methyl acrylate) homopolymer (PMA_97_, *M*_n,NMR_ = 8500 Da, *Đ* = 1.09, Figures S2-2), kinetic studies were conducted on the photopolymerization of these systems (Figure S4). Both monomers demonstrated similarly rapid polymerization rates, with originally linear trends in the plot of ln([M]_0_/[M]) vs reaction time and near complete conversions reached within 2 h. Most importantly, dispersity values were kept low and shown to marginally decrease with the growth of the chains. To further validate the feasibility of our method, we performed *in situ* chain extension tests, producing PMA-*b*-PMA and PBSPA-*b*-PBSPA “homopolymers” (Figure S5). While both macroinitiators could be successfully extended with the same monomer, conversions of only 68% and 85% were reached after 2 h for MA and BSPA, respectively (Tables S1 and S2).

Then, we attempted the *in situ* production of amphiphilic block copolymers (BCPs). While MA and BSPA monomers exhibit similar polymerization kinetics and can be sequentially polymerized in a reserve order (Figure S6-1), we decided to first grow the hydrophobic segment, followed by the protected sulfonate. This would ensure that in the case of incomplete conversion, no anionic monomer unit would be present within the hydrophobic block. In a first attempt, near-quantitative conversion was reached after 2 h for the first block (i.e., PMA), while only 72% was obtained after 16 h for the second one (i.e., PBSPA). We noticed that the reaction mixture had become highly viscous, impeding the rotation of the stirring bar, so another reaction was conducted with the addition of DMSO upon introduction of the BSPA monomer, which led to a minor improvement (84% conversion). In a last attempt, solvent and fresh Me_6_-TREN as well as CuBr_2_ were added upon the introduction of the protected monomer. This led to almost full conversion (98%) after 16 h, enabling the formation of a protected PMA_97_-*b*-PBSPA_98_ (*M*_n,NMR_ = 33000 Da, *Đ* = 1.10) precursor, which was subsequently deprotected *in situ* by the addition of solvent and NaI within the same vessel, yielding a PMA_97_-*b*-PSPA-Na_98_ (*M*_n,NMR_ = 27500, *x*_SPA-Na_ = 0.50) amphiphilic BCP. Characteristics and analyses of these optimization attempts can be found in Table S4 and Figure S6-2. ^1^H NMR (Figure 1A) and SEC (Figure 1B) were used to verify the composition of the optimized system and ensure adequate chain extension and minimization of side reactions. The amphiphilic nature of PMA_97_-*b*-PSPA-Na_98_ (i.e., hydrophobic PMA and hydrophilic/anionic PSPA-Na) imparts an ability to self-assemble in aqueous media. A small amount of salt was added to enable the screening of polyelectrolytes and reduce aggregation (i.e., slow diffusion mode).^33^ The *direct dissolution* method (i.e., an appropriate volume of 10 mM KNO_3_ was directly added into a vial charged with dried BCP to achieve a 1 g L^–1^ concentration), readily permitting the formation of micelles featuring a hydrophobic PMA core and a negatively charged PSPA-Na shell, as evidenced by dynamic light scattering (DLS, Figure S7) and their ζ-potential. Visualization of the nanoparticles was permitted by transmission electron microscopy (TEM, Figures 1 and S8) on negatively stained samples, with the PMA core (i.e., low electron density) appearing clear, while the uranyl acetate stain (i.e., high electron density) penetrates the negatively charged shell of the micelles, appearing dark.

**Figure 1:**
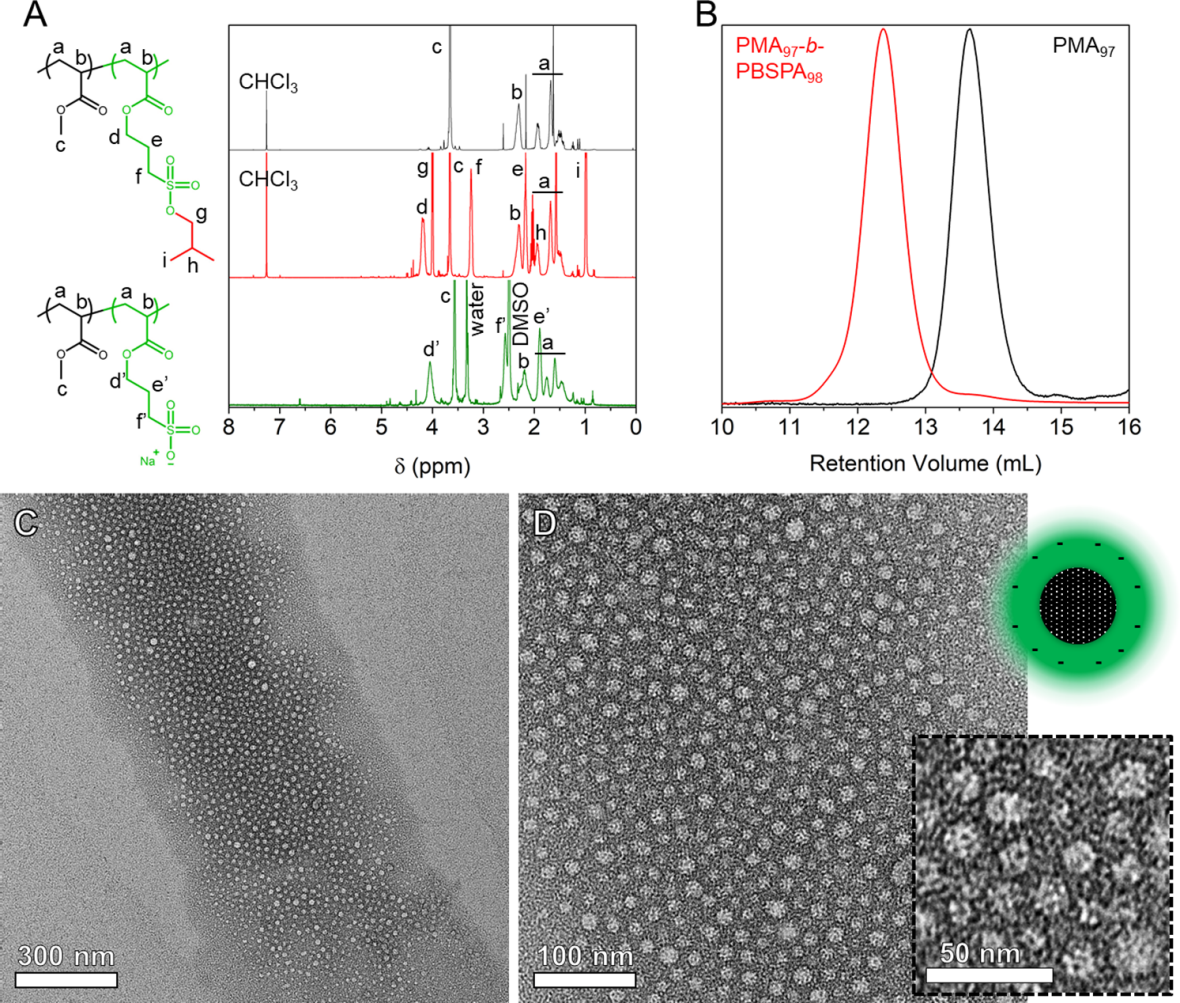
Optimized synthesis of a strong amphiphile in a one-pot fashion and its self-assembly in an aqueous medium. Characterization of the polymer and its precursors (PMA_97_, black, CDCl_3_; PMA_97_-*b*-PBSPA_98_, red, CDCl_3_; PMA_97_-*b*-PSPA-Na_98_, green, DMSO-*d*_6_) by (A) ^1^H NMR and (B) SEC in DMF with 0.01 M LiBr. (C, D) Negatively stained TEM images of nanoparticles achieved through direct dissolution of PMA_97_-*b*-PSPA-Na_98_ at 1 g L^–1^ in 10 mM KNO_3_.

Next, we produced a series of PMA_*x*_-*b*-PSPA-Na_*y*_ amphiphilic BCPs in a one-pot fashion with varying compositions (DP_total_ ∼ 300, *M*_n,NMR_ = 30500–59000 Da, *x*_SPA-Na_ = 0.17–0.84, Table 1). After verifying the polymers’ composition by ^1^H NMR, SEC and Fourier transform infrared spectroscopy (FTIR, Figures S9 and S10), we studied the effect of the SPA-Na molar fraction on their thermal properties, starting with thermogravimetric analysis (TGA, Figure 2A_1_). All protected copolymers are stable up to ∼180 °C, after which they rapidly start degrading, with the loss of the isobutoxy protective groups. The percentage of weight loss between 200 and 400 °C (i.e., acid-catalyzed hydrolysis of the acrylic ester)^21^ and the rate of degradation within this range are dictated by *x*_BSPA_ within the chains, as this block degrades faster than more thermally stable PMA (see comparative thermograms in Figure S11A). Differential scanning calorimetry (DSC, Figure 2B_1_) on the BCP with the lowest *x*_BSPA_ evidenced the presence of two glass transition temperatures (*T*_g1_ = −5.7 °C and *T*_g2_ = 12.4 °C) attributed to PBSPA and PMA segments, respectively (Figure S11B), which would indicate demixing of the blocks. While this behavior is also observed for *x*_BSPA_ = 0.33 and 0.53, BCPs with higher molar fractions display a single *T*_g_ at ∼−8 °C, close to that of the PBSPA homopolymer (Table S9). Small-angle X-ray scattering (SAXS, Figure 2C_1_) analyses on the protected BCPs further evidenced phase separation for BCPs with *x*_BSPA_ = 0.17–0.70, exhibiting only short-range order and an average domain spacing of 2π/q* ≈ 23 nm. When the BSPA molar fraction is 0.84, no signal is monitored, indicating a disordered structure. These SAXS results point toward a small value of the Flory–Huggins interaction parameter for the protected BCPs. Once deprotected, the BCPs become thermally more stable, as evidenced by the higher degradation temperature (i.e., ∼375 °C; Figure 2A_2_), after which both PMA and PSPA-Na segments deteriorate. One noticeable difference lies in the weight percentage of char left at 700 °C, which increases from 22 to 41 wt % when *x*_SPA-Na_ increases, explained by the higher content of inorganic residue. Once deprotected, PSPA-Na does not possess a measurable *T*_g_; consequently, BCPs with large *x*_SPA-Na_ do not exhibit a glass transition temperature either, with the exception of *x*_SPA-Na_ = 0.17, displaying that of the long PMA segment (Figure 2B_2_). As expected, deprotection results in a stronger segregation due to the increased block incompatibility. SAXS analyses of the annealed deprotected polymers (Figure 2D_2_) evidenced the presence of a wide range of structures. Surprisingly, the data obtained from samples with *x*_SPA-Na_ = 0.17 and 0.33 feature Bragg peaks at q*, 2q*, and 3q*, suggesting a lamellar morphology, while hexagonally packed cylinders would be expected from classic theory of BCPs.^34^ This is indicative of strong electrostatic repulsion between the BCP charged segments.^35^ The profile of *x*_SPA-Na_ = 0.33 further shows a broad signal overlapping with the 2q* peak, which could potentially indicate the presence of hexagonal perforation. As the SPA-Na molar fraction increases to 0.53, 0.70, and 0.84, the BCPs’ morphology in bulk appears to change into hex, bcc, and fcc, respectively.^36^ The domain spacings of the deprotected systems are 47, 51, 43, 40, and 33 nm.

**Table 1:** Characteristics of the PMA_*x*_-*b*-PSPA-Na_*y*_ Amphiphilic BCPs Produced in a One-Pot Fashion and PMA_102_-*b*-PSPA-Na_*x*_ Polymers Produced through Sampling

	*x*_SPA-Na_^a^ (mol %)	*M*_n,NMR_^a^ (Da)	*M*_n,SEC_^b^ (Da)	*Đ*^b^	*D*_h_^c^ (nm)	PDI^c^	ζ^c^ (mV)
PMA_97_-*b*-PSPA-Na_98_	50	27500	38700	1.10	185.6 ± 12.0	0.529 ± 0.162	–35.4 ± 2.4
PMA_231_-*b*-PSPA-Na_48_	17	30500	50500	1.17	114.3 ± 2.6	0.331 ± 0.032	–32.2 ± 1.4
PMA_193_-*b*-PSPA-Na_94_	33	37100	51800	1.18	122.1 ± 1.4	0.234 ± 0.016	–23.7 ± 1.9
PMA_146_-*b*-PSPA-Na_164_	53	48200	58100	1.15	141.2 ± 7.0	0.359 ± 0.032	–40.7 ± 0.2
PMA_98_-*b*-PSPA-Na_228_	70	57800	68300	1.17	223.2 ± 2.2	0.289 ± 0.029	–30.9 ± 1.3
PMA_47_-*b*-PSPA-Na_254_	84	59100	90100	1.18	302.3 ± 27.5	0.436 ± 0.088	–43.1 ± 0.9
PMA_102_	n.a.	9000	11400	1.10	n.a.	n.a.	n.a.
PMA_102_-*b*-PSPA-Na_93_	48	29000	39100	1.10	103.8 ± 1.9	0.300 ± 0.025	–42.4 ± 0.9
PMA_102_-*b*-PSPA-Na_140_	58	39200	48300	1.10	68.7 ± 5.6	0.290 ± 0.032	–44.0 ± 1.1
PMA_102_-*b*-PSPA-Na_207_	67	53700	65200	1.14	104.6 ± 0.9	0.333 ± 0.013	–41.3 ± 1.1

a Determined by ^1^H NMR from a combination of conversion samples and end-group analysis.

b Determined by SEC in DMF with 0.01 M LiBr on the purified protected intermediate and calibrated against near-monodisperse PMMA standards.

c Determined by DLS/ζ-potential measurements at 25 °C on 1 g L^–1^ solutions in 10 mM KNO_3_ and measured in triplicate.

**Figure 2:**
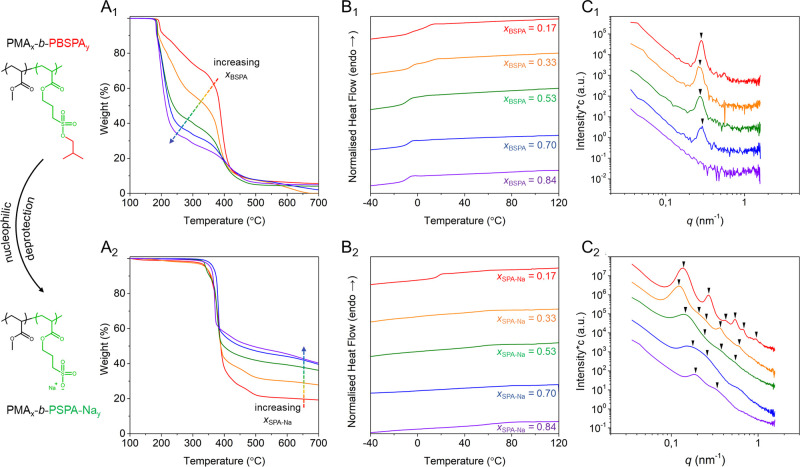
Comparative thermal and scattering analyses of the block copolymers produced in this study. TGA of (A_1_) protected and (A_2_) deprotected BCPs, DSC thermograms of (B_1_) protected and (B_2_) deprotected BCPs, and SAXS profiles of (C_1_) protected and (C_2_) deprotected BCPs measured in bulk after annealing. Note that SAXS curves have been offset vertically for clarity. Polymers are PMA_231_-*b*-P(B)SPA_48_ (*x* = 0.17, red), PMA_193_-*b*-P(B)SPA_94_ (*x* = 0.33, orange), PMA_146_-*b*-P(B)SPA_164_ (*x* = 0.53, green), PMA_98_-*b*-P(B)SPA_228_ (*x* = 0.70, blue), and PMA_47_-*b*-P(B)SPA_254_ (*x* = 0.84, violet). (C_1_) Black ticks indicate short-range ordering with a corresponding Bragg peak at q*. (C_2_) Black ticks indicate lamellar phase structure with q*, 2q*, 3q*, 4q*, 5q*, and 7q* peak ratios for PMA_231_-*b*-PSPA-Na_48_; lamellar phase with q*, 2q*, 3q*, 4q*, and 5q* peaks for PMA_193_-*b*-PSPA-Na_94_, along with the √3q* peak indicative of hexagonal perforations; hexagonal phase with q*, √3q*, √7q*, and √13q* peaks for PMA_146_-*b*-PSPA-Na_164_; body-centered cubic phase with q*, √2q*, and 4q* peaks for PMA_98_-*b*-PSPA-Na_228_; face-centered cubic phase with q* and √3q* peaks for PMA_47_-*b*-PSPA-Na_254_.

The amphiphilic nature of the PMA_*x*_-*b*-PSPA-Na_*y*_ BCPs permits their self-assembly in aqueous media. DLS and ζ-potential measurements (Figure 3A_1_**–**E_1_ and Table 1) confirmed the presence of nanoparticles with mean hydrodynamic diameters ranging from *D*_h_ = 122 to 302 nm and stabilized by a negatively charged shell, while negatively stained TEM images (Figure 3A_2_**–**E_2_ and Supporting Information S12) evidenced the presence of micelles. Note that average *D*_h_ values were marginally larger than one could expect, likely originating from the presence of a few larger specimens, as observed in TEM images. Nonetheless, spherical micelles were observed for all BCPs, despite the large span of their *x*_SPA-Na_ molar fractions (*x*_SPA-Na_ = 0.17–0.84). Mean core diameter values (ϕ_core_ = 12.2–19.1 nm, Figure S12-6) were extracted from the TEM images through statistical analysis, with particles obtained from BCPs with lower *x*_SPA-Na_ having larger cores compared to that produced from BCPs with higher *x*_SPA-Na_ values. SAXS measurements (Figure 3A_3_–E_3_ and Figure S13) were also conducted on self-assembled nanoparticles in solution. The profiles are compatible with the presence of spherical nanoparticles and can be fitted using a spherical core–shell model with a core size polydispersity. The first minimum of the SAXS curves, which is indicative of the average size of the nanoparticles, shifts toward low *q* values (i.e., larger particle radius) when increasing the molar fraction of PSPA-Na. Generally, the total size of the particles is smaller than the overestimated values derived by DLS,^37^ but this is expected, as the large negative charge of the corona introduces large electrostatic repulsion, thus providing large hydrodynamic radii. The structural parameters derived from the SAXS data fitting are reported and commented on in detail in Supporting Information S13.

**Figure 3:**
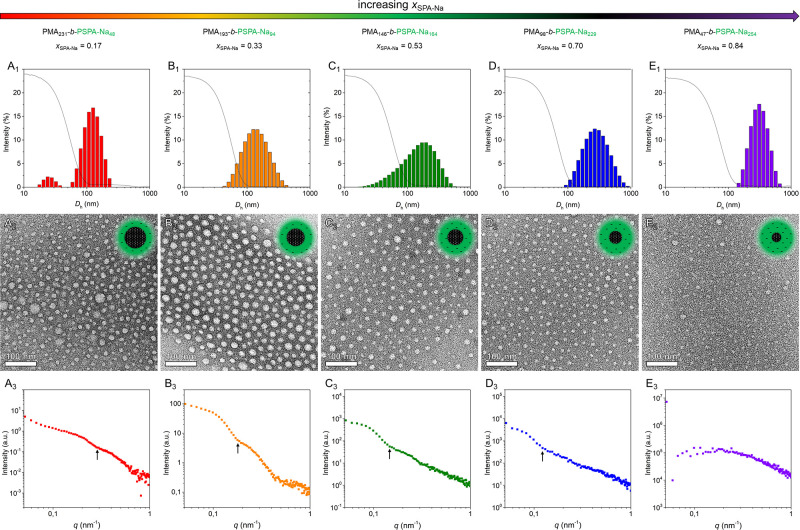
Comparative self-assembly of the amphiphilic block copolymers in aqueous media. (A1–E1) DLS intensity plots (bars) and correlogram functions (solid lines), (A2–E2) negatively stained TEM images, and (A3–E3) small-angle X-ray scattering plots measured in solution. The black arrows in the SAXS profile depict the minimums when available.

Finally, to expand the scope of our study, we produced three BCPs in a one-pot fashion using a sampling technique (Figure S14). First, the hydrophobic block (PMA_102_, *M*_n,NMR_ = 9000 Da, *Đ* = 1.10) was polymerized using the photoreactor and further chain extended with BSPA monomer. During the growth of the second block, large aliquots were withdrawn under an inert atmosphere and dispensed into individual glass vials alongside solvent and NaI for deprotection. ^1^H NMR (Figure S15) and SEC (Figure 4A) performed on the aliquots (i.e., PMA_102_-*b*-PBSPA_*x*_ protected block copolymers, *x* = 93, 140, or 207) permitted the determination of the block copolymer composition and the verification of the successful chain extension. After nucleophilic deprotection and purification, a series of amphiphilic BCPs with consistent hydrophobic blocks yet varying polyanionic molar ratios (*x*_SPA-Na_ = 0.48–0.67) were obtained. Owing to their amphiphilic nature, the three PMA_102_-*b*-PSPA-Na_*x*_ block copolymers were able to spontaneously self-assemble into micelles when dispersed in aqueous media, as evidenced by DLS (Figure S16 and Table 1) and visualized by negatively stained TEM (Figures 4B and S17).

**Figure 4:**
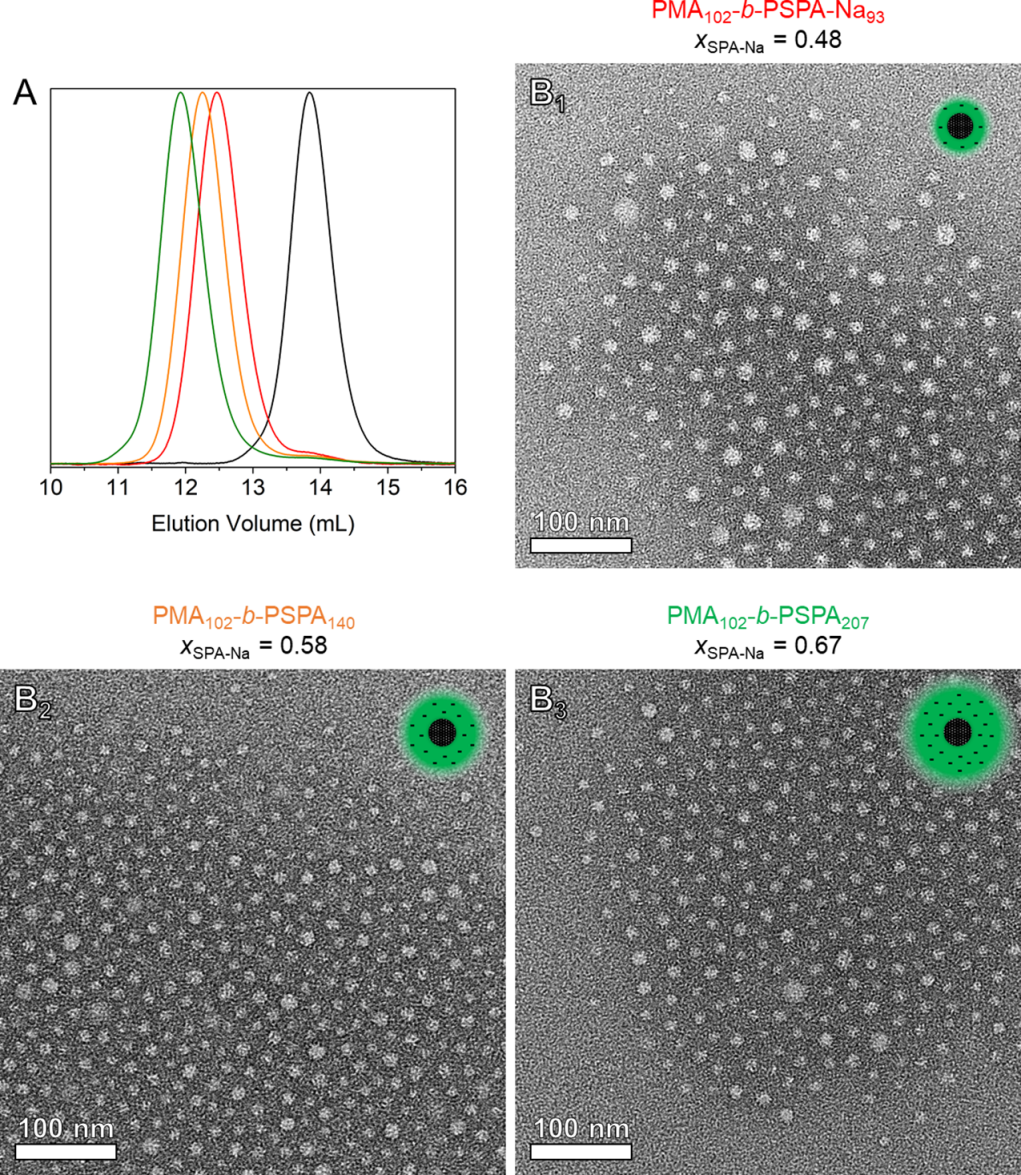
Synthesis of several amphiphilic block copolymers by sampling a single one-pot polymerization reaction. (A) SEC elugrams of PMA_102_ (black), PMA_102_-*b*-PBSPA_93_ (red), PMA_102_-*b*-PBSPA_140_ (orange), and PMA_102_-*b*-PBSPA_207_ (green). (B) Negatively stained TEM images of nanoparticles achieved from the self-assembly of BCPs at 1 g L^–1^ in 10 mM KNO_3_.

Herein, we developed a methodology for the straightforward synthesis of strong anionic and charge-neutral block copolymers in a one-pot fashion. Photo-ATRP enables the rapid growth of a hydrophobic macroinitiator, followed by *in situ* chain extension with a protected sulfonate monomer and subsequent nucleophilic deprotection. A series of poly(methyl acrylate)-*block*-poly(sulfopropyl acrylate sodium salt) amphiphilic BCPs with varying compositions (*x*_SPA-Na_ = 0.17–0.84) were produced through a simple change in the monomer feed ratio. While the protected precursors are fully hydrophobic, which facilitates their characterization, the deprotected macromolecules are capable of self-assembly into micelles in aqueous media. A sampling methodology further enabled the facile production of copolymers with a constant hydrophobic block but varying hydrophilic segments. Our synthesis routes pave the way for the streamlined synthesis of sequence-controlled macromolecules with chemistries that are challenging or time-consuming to include otherwise.

## Supporting Information Available

Materials and methods as well as additional ^1^H NMR spectra, SEC elugrams, DSC, and TGA thermograms, DLS plots, TEM images, and SAXS profiles (PDF)
