# Supplementary material for: One-Pot Synthesis of Strong Anionic/Charge-Neutral Amphiphilic Block Copolymers
Source: ACS Macro Lett. 2023 Jul 18;12(8):1071–8. doi: 10.1021/acsmacrolett.3c00355 (PMC10433517; doi:10.1021/acsmacrolett.3c00355)
Supplement: Supplementary file 1 — mz3c00355_si_001.pdf [file mz3c00355_si_001.pdf]

# One-Pot Synthesis of Strong Anionic/Charge-Neutral Amphiphilic Block Copolymers

Théophile Pelras*[a], András Eisenga[a,b], Gábor Érsek[c], Aldo Altomare[a], Giuseppe Portale[c], Marleen Kamperman[b] and Katja Loos*[a]

[a] Macromolecular Chemistry and New Polymeric Materials, Zernike Institute for Advanced Materials, University of Groningen, Nijenborgh 4, 9747 AG, Groningen, The Netherlands

[b] Polymer Science, Zernike Institute for Advanced Materials, University of Groningen, Nijenborgh 4, 9747 AG, Groningen, The Netherlands

[c] Physical Chemistry of Polymeric and Nanostructured Materials, Zernike Institute for Advanced Materials, University of Groningen, Nijenborgh 4, 9747 AG, Groningen, The Netherlands

# Supporting Information

# Contents


Materials ........ 3
Methods ........ 4
Polymer Synthesis ........ 7
Supplementary Figures ........ 20
S1: Photographs. ........ 20
S2: Photo-RDRP of homopolymers. ........ 21
S3: Deprotection of isobutoxy-protected homopolymer. ........ 22
S4: Kinetics of photo-RDRP homopolymerizations. ........ 23
S5: Chain extension tests on PMA and PBSPA homopolymers. ........ 24
S6: Optimization of the one-pot polymerization of $PMA_x$-b-PSPA-$Na_y$. ........ 25
S7: DLS of nanoparticles of the optimized block copolymer. ........ 27
S8: Electron microscopy images of nanoparticles of the optimized block copolymer. ........ 28
S9: $^1$H NMR and SEC analyses of the $PMA_x$-*b*-PSPA-$Na_y$ block copolymers. ........ 29
S10: Infrared spectroscopy of homopolymers and block copolymers. ........ 32
S11: Thermal analyses of homo- and block copolymers. ........ 34
S12: Electron microscopy images of the $PMA_x$-*b*-PSPA-$Na_y$-based nanoparticles. ........ 36
S13: SAXS of the polymer micelles in aqueous solution. ........ 40
S14: Schematic representation of the 'sampling' method. ........ 42
S15: $^1$H NMR of the block copolymers achieved through sampling. ........ 43
S16: DLS analyses of the amphiphilic BCPs produced through sampling. ........ 44
S17: Electron microscopy images of the amphiphilic BCPs produced through sampling. ........ 45
Supporting References ........ 47

## Materials

The monomer 3-isobutoxysulfopropyl acrylate was synthesized as reported before.[1] Aluminum oxide (AlOx, basic, activated), copper(II) bromide ($CuBr_2$, 99%), 1,4-dioxane (99.8 %), ethyl α-bromoisobutyrate (EB*i*B. 98 %), methyl acrylate (MA, 99 %) and tris[2-(dimethylamino)ethyl]amine ($Me_6$-TREN, 97 %) were purchased from Sigma-Aldrich. *n*-hexane (HPLC grade) was obtained from Macron Fine Chemicals. Absolute ethanol (99.9 %) and dimethyl sulfoxide (DMSO, 99.9 %) were purchased from J.T. Baker. Sodium iodide (NaI, ACS reagent grade) was purchased from Acros Organics.

Commercially-available monomers were passed through a short AlOx column to remove inhibitors prior to polymerizations. All other chemicals were used as received.

$Me_6$-TREN/$CuBr_2$ stock solutions were prepared freshly prior to polymerization by introducing 1 eq $CuBr_2$, 6 eq $Me_6$-TREN and DMSO into a glass vial and thorough mixing. A calculated volume of the stock solution was introduced into the reaction mixtures to obtain 0.02 eq $CuBr_2$ and 0.12 eq $Me_6$-TREN, with respect to the initiator. An example of a stock solution is as follows: $CuBr_2$ (1 eq, 4.52 mg, 20.3 μmol), $Me_6$-TREN (6 eq, 28.1 mg, 122 μmol) and DMSO (5.07 mL). A volume of 128 μL of this stock solution is required for a reaction using 5.00 mg EB*i*B.

The light reactor was built using a 2 m LED strip (SimpleColor Blue, purchased from Waveform) emitting at 365 nm with an output of ~ 29 W (original length of 5 m for 72 W) winded inside a Ø14 cm crystallization dish, which was covered on the exterior with aluminum foil with its reflective side inward (see **Figure S1**). Note that no active cooling was required, as the temperature inside the reactor never exceeded 35 °C during the course of the reactions.

# Methods

**Proton nuclear magnetic resonance ($^1$H NMR)** spectra were recorded on an Agilent 400-MR 400 MHz spectrometer at 298 K. Deuterated chloroform ($CDCl_3$, 99.8 %), deuterated dimethyl sulfoxide (DMSO-$d_6$, 99.9 %) and deuterium oxide ($D_2O$, 99.9 %) were purchased from Sigma-Aldrich. Samples were dissolved in an appropriate solvent or solvent mixture (≈ 5 g $L^{-1}$) and analyzed with a pulse width of 45 μs, spectral width of 12/-2 ppm, recycle delay of 1 s and either 32 or 256 scans (conversion or purified samples respectively). Spectra were analyzed with Mestrenova software version 14.1.

**Size-exclusion chromatography (SEC)** was performed on a GPCMax system from Viscotek equipped with a 302 TDA detector array and two columns in series (PolarGel L and M, both 8 μm 30 cm) from Agilent Technologies. The columns and detectors were maintained at a temperature of 50 °C. DMF containing 0.01 M LiBr was used as the eluent at a flow rate of 1 mL $min^{-1}$. Near monodisperse poly(methyl methacrylate) standards from Polymer Standard Services were used for the construction of a calibration curve. Samples were dissolved in the eluent at a concentration of ≈ 3 g $L^{-1}$ and passed through a 0.45 μm PTFE filter prior to injection. Data acquisition and calculations were performed using Viscotek Omnisec software version 5.0.

**Attenuated total reflection–Fourier transform infrared (ATR-FTIR)** spectra were recorded on a Bruker VERTEX 70 spectrometer equipped with an ATR diamond single reflection module. The spectra were collected in the range of 4000–500 $cm^{-1}$ with a spectral resolution of 2 $cm^{-1}$ and using 64 scans for each sample. Atmospheric compensation and baseline correction were applied to the collected spectra using Bruker's OPUS spectroscopy software version 7.0.

**Differential scanning calorimetry (DSC)** measurements were recorded on a TA Instruments DSCQ1000 analyzer. The samples (~ 5 mg) were subjected to the following method: (i) equilibration at - 60 °C, (ii) 5 min isotherm, (iii) ramp to 130 °C at 10 °C $min^{-1}$, (iv) 5 min isotherm, (v) ramp to - 60 °C at 10 °C $min^{-1}$, (vi) 5 min isotherm and (vii) ramp to 130 °C at 10 °C $min^{-1}$. Data analysis was performed on the heat second cycle using TA Instruments TRIOS software.

**Thermogravimetric analysis (TGA)** measurements were recorded on a TA Instruments TGA5500 analyzer. The samples (~ 5 mg) were heated from 30 °C to 700 °C at a rate of 10 °C $min^{-1}$ under a continuous nitrogen flow. The data acquisition and analysis was done using TA Instruments TRIOS software.

**Dynamic light scattering (DLS)** measurements were performed on a Malvern Panalytical Zetasizer Ultra system, equipped with a helium-neon laser (λ = 633 nm) and an Avalanche photodiode detector. The nanoparticles solutions were measured at 25 °C in back scattering mode after a 120 s equilibration time and using 30 cumulative recordings. Samples were recorded in triplicate. The results were analyzed with ZS Xplorer software.

**ζ-potential** measurements were performed on a Malvern Panalytical Zetasizer Ultra system, equipped with a helium-neon laser (λ = 633 nm) and an Avalanche photodiode detector. The measurements were taken at 25 °C while the acquisition times were determined automatically. Samples were recorded in triplicate and the results were analyzed with ZS Xplorer software.

**Transmission electron microscopy (TEM)** imaging was performed on a Philips CM120 transmission electron microscope using a tungsten filament operated at an accelerating voltage of 120 kV. Images were recorded using a Gatan 4k CCD camera. TEM grids (carbon, 400 mesh with carbon support film) were glow-discharged (15 s at 50 mA and 300 V) prior to sample preparation. Specimens were prepared by deposition of 5 µL of the nanoparticle dispersion (c ~ 1 g $L^{-1}$) onto the grid and adsorption for 1 min before blotting. Before the specimen was fully dried, 5 µL of 2 wt.% uranyl acetate staining solution was deposited onto the grid, immediately blotted and a new 5 µL drop of staining solution was deposited and left to adsorb for 1 min before blotting. TEM images were analyzed using Image J software, employing brightness and contrast correction tools to enhance the general quality of the snapshots, and software-imbedded measurement tool to determine the dimensions of the nanoparticles.

**Small angle X-ray scattering (SAXS)** experiments were performed at the MINA diffractometer of the University of Groningen. The instrument was equipped with a Cu rotating anode (X-ray wavelength λ = 1.5413 Å and energy of 8 keV) and a Dectris Pilatus 300K detector placed at a distance of 3.1 m. SAXS measurements of the block copolymers in bulk were conducted after annealing at 120 °C overnight (protected samples) or for a week (deprotected samples) and using a copper sample holder sealed with Kapton™ tape. The solution scattering experiments were carried out in glass capillaries, using 5 g $L^{-1}$ solutions. The data were fitted with SASfit software.[2] The scattering length densities η (SLD) were computed using the nominal composition of the polymers according to the synthesis procedure. The SLD for the solvent at room temperature was used as $\eta_m = 0.94 \times 10^{11}$ $cm^{-2}$. The contrast between the core and the solvent ($\eta_{core}$-$\eta_m$) was calculated as $0.16 \times 10^{11}$ $cm^{-2}$ and kept constant during the fitting procedure. The SAXS profiles for the polymer micelles were fitted using a dilute core-shell model for spherical particles. In this case, the scattering intensity is given by the following equation:

$$I_p(q) = [P(q, R_{core} + R_{shell}, (\eta_{shell} - \eta_{solvent})) - P(q, R_{core}, (\eta_{shell} - \eta_{core}))]^2$$

with:

$$P(q, R, \eta) = \frac{4}{3}\pi R^3 \eta * 3 \frac{sin(qR) - qRcos(qR)}{(qR)^3}$$

where $R_i$ and $\eta_i$ are the size and scattering length density for the core and shell according to the

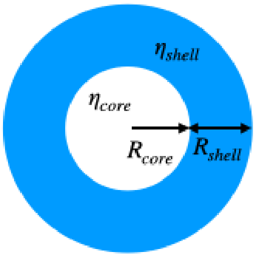


scheme below:

An additional background with equation $I_{bkg} = a + bq^{-\alpha}$ was also considered to take into account the extra contributions in the sample. For instance, the $PMA_{231}$-*b*-PSPA-$Na_{48}$ clearly shows an intensity rise at low q-values that cannot be attributed to the particle form factor and may evidence some particle aggregation, or the presence of few very large particles. The total experimental intensity is thus fitted as follows:

$$I(q) = I_p(q) + I_{bkg}$$

**Solution self-assembly** of the amphiphilic block copolymers was achieved through the ‘direct dissolution’ method, *i.e.*, the dry material was straightforwardly dissolved in the solution without prior treatment. With the exception of $PMA_{231}$-*b*-$PSPA_{48}$, solutions were prepared by weighing a few milligrams of polymer and adding an appropriate amount of triple-filtered (0.2 µm cellulose acetate) 10 mM $KNO_3$ solution to achieve a 1 g $L^{-1}$ concentration. For $PMA_{231}$-*b*-$PSPA_{48}$, the polymer was first dissolved in 10 vol.% triple-filtered DMSO, then 10 vol.% 1:1 triple-filtered DMSO:water was introduced upon stirring before 80 vol.% 10 mM $KNO_3$ was added (final polymer concentration: 1 g $L^{-1}$, 8 mM $KNO_3$ in 15:85 DMSO:water). All solutions were stirred for a few days before they were heated to their boiling point.

# Polymer Synthesis

### Photo-RDRP of methyl acrylate.

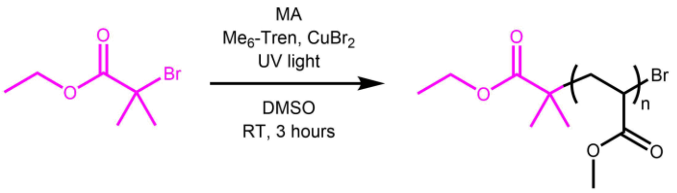


EB*i*B (1 eq, 40.6 mg, 208 μmol), MA (100 eq, 1798 mg, 20.9 mmol), $Me_6$-TREN (0.12 eq, 5.88 mg 25.5 μmol, 1.07 mL of a stock solution of 269 mg $Me_6$-TREN in 4.94 mL DMSO), $CuBr_2$ (0.02 eq, 0.90 mg, 4.04 μmol, 1.07 mL of a stock solution of 4.11 mg $CuBr_2$ in 4.94 mL DMSO) and DMSO (200 eq, 1.8 mL, total volume of 2.9 mL) were charged into a Schlenk tube equipped with a stirring egg. The reaction mixture was deoxygenated via 3 freeze-pump-thaw cycles and backfilled with argon before an aliquot was withdrawn under argon protection for $^1$H NMR sampling. The tube was then placed inside the light reactor and after 3 hours, the light was switched off and the Schlenk tube was opened to air. After withdrawal of an aliquot for $^1$H NMR sampling, the reaction mixture was precipitated in cold 6:1 *n*-hexane:ethanol. The polymer was redissolved in THF and precipitated once more in cold 6:1 *n*-hexane:ethanol. The polymer was redissolved in minimal 1,4-dioxane and freeze-dried overnight to yield a transparent highly viscous liquid. Yield: 1.37 g. $^1$H NMR: conversion = 97 %, $DP_{NMR}$ = 97, $M_{n\ NMR}$ = 8 500 Da. SEC: $M_{n\ SEC}$ = 10 700 Da, Đ = 1.09.

**Photo-RDRP of 3-isobutoxysulfopropyl acrylate.**

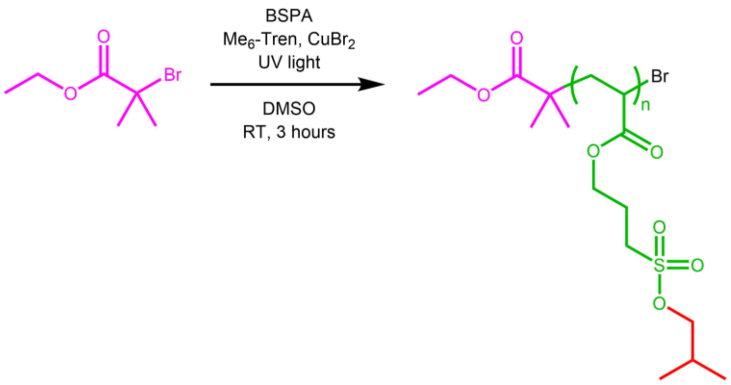


EB*i*B (1 eq, 4.96 mg, 25.4 µmol, 138 µL of a stock solution of 109 mg EB*i*B in 3.02 mL DMSO), BSPA (104 eq, 659 mg, 2.63 mmol), $Me_6$-TREN (0.12 eq, 0.72 mg 3.10 µmol, 175 µL of a stock solution of 13.5 mg $Me_6$-TREN in 2.03 mL DMSO), $CuBr_2$ (0.02 eq, 0.11 mg; 0.51 µmol, 175 µL of a stock solution of 2.21 mg $CuBr_2$ in 2.03 mL DMSO) and DMSO (200 eq, 56 µL, total volume of 369 mL) were charged into a Schlenk tube equipped with a stirring egg. The reaction mixture was deoxygenated via argon bubbling for 5 min before an aliquot was withdrawn under argon protection for $^1$H NMR sampling. The tube was then placed inside the light reactor and after 3 hours, the light was switched off and the Schlenk tube was opened to air. After withdrawal of an aliquot for $^1$H NMR sampling, the reaction mixture was precipitated in cold 6:1 *n*-hexane:ethanol. The polymer was redissolved in THF and precipitated once more in cold 6:1 *n*-hexane:ethanol. The polymer was redissolved in minimal 1,4-dioxane and freeze-dried overnight to yield a transparent highly viscous liquid. Yield: 578 mg. $^1$H NMR: conversion = 99 %, $DP_{NMR}$ = 99, $M_{n\ NMR}$ = 24 900 Da. SEC: $M_{n\ SEC}$ = 35 500 Da, Đ = 1.12.

**Deprotection of poly(3-isobutoxysulfopropyl acrylate).**

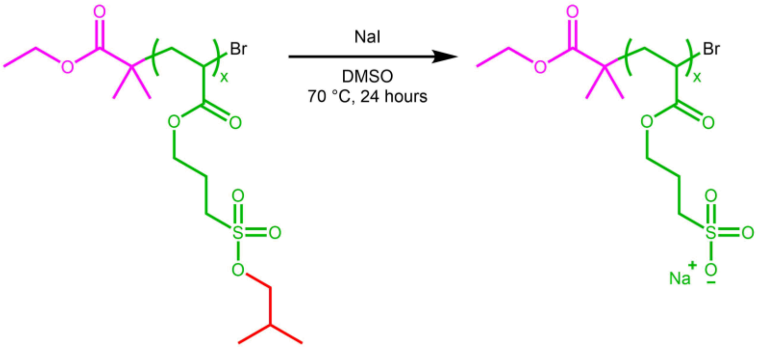


The deprotection of poly(3-isobutoxysulfopropyl acrylate) was performed as reported before.[1] $PBSPA_{99}$ (1 eq, 103 mg, 412 µmol BSPA) and NaI (3 eq per BSPA unit, 183 mg, 1.22 mmol) were dissolved in 3 mL DMSO and charged into a glass vial equipped with a stirring bar. The reaction mixture was stirred at 70 °C for 24 hours. The resulting dark brown solution was precipitated in *n*-hexane:ethanol 1:2, washed several times with *n*-hexane:ethanol 1:1 until the supernatant became colourless before a final wash with pure *n*-hexane. The polymer was redissolved in minimal DI water and freeze-dried overnight to yield an off-white brittle solid. Yield: 62 mg. $^1$H NMR: deprotection ≈ 100 %, $M_{n\ NMR}$ = 21 600 Da.

**Kinetic study of the photo-RDRP of methyl acrylate.**

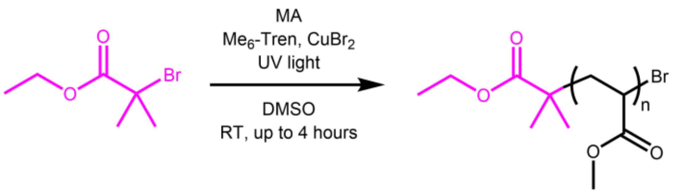


EB*i*B (1 eq, 5.03 mg, 25.8 µmol, 138 µL of a stock solution of 109 mg EB*i*B in 3.02 mL DMSO), MA (102 eq, 226 mg, 2.63 mmol), $Me_6$-TREN (0.12 eq, 0.72 mg, 3.1 µmol, 70 µL of a stock solution of 20.0 mg $Me_6$-TREN in 2.02 mL DMSO), $CuBr_2$ (0.02 eq, 0.12 mg, 0.54 µmol, 70 µL of a stock solution of 3.35 mg $CuBr_2$ in 2.02 mL DMSO) and DMSO (200 eq, 161 µL, total volume of 369 µL) were charged into a Schlenk tube equipped with a stirring egg. The reaction mixture was deoxygenated via 3 freeze-pump-thaw cycles and backfilled with argon before an aliquot was withdrawn under argon protection for $^1$H NMR sampling. The tube was then placed inside the light reactor for up to 4 hours. Aliquots were withdrawn with an argon-flushed syringe at pre-set time intervals under argon protection. $^1$H NMR samples (~ 2 drops) were directly diluted in $CDCl_3$ while SEC samples (~ 2 drops) were precipitated in  cold 6:1 *n*-hexane:ethanol, dried in air and dissolved in eluent.

**Kinetic study of the photo-RDRP of 3-isobutoxysulfopropyl acrylate.**

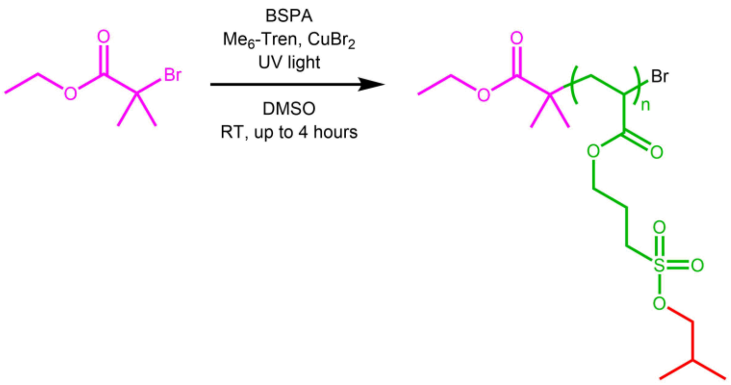


EB*i*B (1 eq, 5.02 mg, 25.7 µmol, 138 µL of a stock solution of 109 mg EB*i*B in 3.02 mL DMSO), BSPA (99 eq, 636 mg, 2.54 mmol), $Me_6$-TREN (0.12 eq, 0.72 mg 3.10 µmol, 61 µL of a stock solution of 22.6 mg $Me_6$-TREN in 1.99 mL DMSO), $CuBr_2$ (0.02 eq, 0.11 mg; 0.51 µmol, 61 µL of a stock solution of 3.60 mg $CuBr_2$ in 1.99 mL DMSO) and DMSO (200 eq, 170 µL, total volume of 369 mL) were charged into a Schlenk tube equipped with a stirring egg. The reaction mixture was deoxygenated via argon bubbling for 5 min before an aliquot was withdrawn under argon protection for $^1$H NMR sampling. The tube was then placed inside the light reactor for up to 4 hours. Aliquots were withdrawn with an argon-flushed syringe at pre-set time intervals under argon protection. $^1$H NMR samples (~ 2 drops) were directly diluted in $CDCl_3$ while SEC samples (~ 2 drops) were precipitated in  cold 6:1 *n*-hexane:ethanol, dried in air and dissolved in eluent.

***In-situ* chain extension of poly(methyl acrylate).**

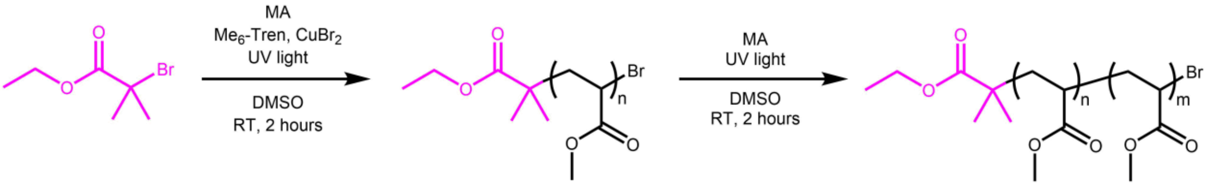


EB*i*B (1 eq, 4.99 mg, 25.6 μmol, 138 μL of a stock solution of 109 mg EB*i*B in 3.02 mL DMSO), MA (103 eq, 227 mg, 2.63 mmol), $Me_6$-TREN (0.12 eq, 0.72 mg 3.1 μmol, 33 μL of a stock solution of 42.2 mg $Me_6$-TREN in 2.05 mL DMSO), $CuBr_2$ (0.02 eq, 0.12 mg, 0.54 μmol, 33 mL of a stock solution of 6.93 mg $CuBr_2$ in 2.05 mL DMSO) and DMSO (200 eq, 198 μL, total volume of 369 μL) were charged into a Schlenk tube equipped with a stirring egg. The reaction mixture was deoxygenated via 3 freeze-pump-thaw cycles and backfilled with argon before an aliquot was withdrawn under argon protection for $^1$H NMR sampling. The tube was then placed inside the light reactor and after 2 hours, the light was switched off and aliquots for $^1$H NMR and SEC analyses (~ 2 drops each) were withdrawn with an argon-flushed syringe. In a separate Schlenk tube, a solution of MA (103 eq, 227 mg, 2.63 mmol) and DMSO (200 eq, 369 μL) was deoxygenated via 3 freeze-pump-thaw cycles and backfilled with argon, before being withdrawn using an argon-flushed syringe and injected into the main Schlenk tube under argon protection. The solution was left to thoroughly mix for a few minutes before an aliquot for $^1$H NMR analysis (~ 2 drops) was withdrawn with a new argon-flushed syringe. The light of the photoreactor was switched back on, and the reaction mixture was stirred for 2 more hours, after which the light was switched off and the Schlenk tube was opened to air. After withdrawal of aliquots for $^1$H NMR and SEC sampling (~ 2 drops each), the reaction mixture was precipitated in cold 6:1 *n*-hexane:ethanol. The polymer was redissolved in THF and precipitated once more in cold 6:1 *n*-hexane:ethanol. The polymer was redissolved in minimal 1,4-dioxane and freeze-dried overnight to yield a transparent highly viscous liquid. Yield: 270 mg. See **Table S1** below for the characterization of the blocks.

**Table S1:** *In-situ* chain extension of poly(methyl acrylate)

| | reaction time (h) | conv.‡ (%) | $M_{n\ NMR}$‡ (Da) | $M_{n\ SEC}$≠ (Da) | Đ≠ |
|---|---|---|---|---|---|
| $PMA_{99}$ | 2 | 96 | 8 700 | 11 500 | 1.12 |
| $PMA_{99}$-*b*-$PMA_{87}$ | 2 | 85 | 16 200 | 18 400 | 1.11 |

‡ determined by $^1$H NMR by comparison of acrylic vs. DMSO signals, ≠ determined by SEC in DMF with 0.01 M LiBr at 50 °C and calibrated against near-monodisperse PMMA.

***In-situ* chain extension of poly(3-isobutoxysulfopropyl acrylate).**

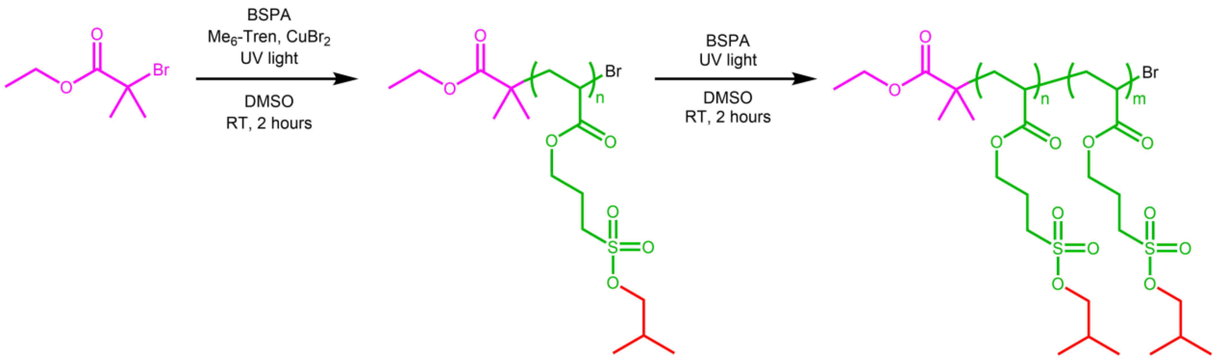


EB*i*B (1 eq, 4.92 mg, 25.2 μmol, 138 μL of a stock solution of 109 mg EB*i*B in 3.02 mL DMSO), BSPA (104 eq, 655 mg, 2.62 mmol), $Me_6$-TREN (0.13 eq, 0.75 mg 3.2 μmol, 53 μL of a stock solution of 24.9 mg $Me_6$-TREN in 2.08 mL DMSO), $CuBr_2$ (0.02 eq, 0.12 mg, 0.54 μmol, 53 mL of a stock solution of 3.96 mg $CuBr_2$ in 2.08 mL DMSO) and DMSO (200 eq, 173 μL, total volume of 369 μL) were charged into a Schlenk tube equipped with a stirring egg. The reaction mixture was deoxygenated via argon bubbling for 5 min before an aliquot was withdrawn under argon protection for $^1$H NMR sampling. The tube was then placed inside the light reactor and after 2 hours, the light was switched off and aliquots for $^1$H NMR and SEC analyses (~ 2 drops each) were withdrawn with an argon-flushed syringe. In a separate Schlenk tube, a solution of BSPA (103 eq, 648 mg, 2.59 mmol) and DMSO (200 eq, 369 μL) was deoxygenated via argon bubbling for 5 min, before being withdrawn using an argon-flushed syringe and injected into the main Schlenk tube under argon protection. The solution was left to thoroughly mix for a few minutes before an aliquot for $^1$H NMR analysis (~ 2 drops) was withdrawn with a new argon-flushed syringe. The light of the photoreactor was switched back on and the reaction mixture was stirred for 2 more hours, after which the light was switched off and the Schlenk tube was opened to air. After withdrawal of aliquots for $^1$H NMR and SEC sampling (~ 2 drops each), the reaction mixture was precipitated in cold 6:1 *n*-hexane:ethanol. The polymer was redissolved in THF and precipitated once more in cold 6:1 *n*-hexane:ethanol. The polymer was redissolved in minimal 1,4-dioxane and freeze-dried overnight to yield a transparent highly viscous liquid. Yield: 860 mg. See **Table S2** below for the characterization of the blocks.

**Table S2:** *In-situ* chain extension of poly(3-isobutoxysulfopropyl acrylate)

| | reaction time (h) | conv.‡ (%) | $M_{n\ NMR}$‡ (Da) | $M_{n\ SEC}$≠ (Da) | Đ≠ |
|---|---|---|---|---|---|
| $PBSPA_{103}$ | 2 | 99 | 25 900 | 43 200 | 1.13 |
| $PBSPA_{103}$-*b*-$PBSPA_{70}$ | 2 | 68 | 43 400 | 60 600 | 1.15 |

‡ determined by $^1$H NMR by comparison of acrylic vs. DMSO signals, ≠ determined by SEC in DMF with 0.01 M LiBr at 50 °C and calibrated against near-monodisperse PMMA.

**Optimization of the one-pot polymerization of poly(methyl acrylate)-*block*-poly(3-isobutoxysulfopropyl acrylate).**

This section details the conditions tested for the production of $PMA_x$-*b*-$PSPA_y$ amphiphilic block copolymers in one-pot fashion. For the methodology, please refer to the following section.

**Table S3:** Compositions of the reaction mixtures used for optimizing the synthesis of $PMA_x$-*b*-PSPA-$Na_y$ block copolymers in one-pot.

| | block 1 | | | | block 2 | | |
|---|---|---|---|---|---|---|---|
| | EB*i*B (μmol) | MA (mmol) | $Me_6$-TREN (μmol) | $CuBr_2$ (μmol) | BSPA (mmol) | $Me_6$-TREN (μmol) | $CuBr_2$ (μmol) |
| $PMA_{113}$-*b*-PSPA-$Na_{72}$ | 25.8 | 3.00 | 2.93 | 0.54 | 2.59 | n.a. | n.a. |
| $PMA_{109}$-*b*-PSPA-$Na_{86}$‡ | 26.1 | 2.94 | 3.37 | 0.53 | 2.63 | n.a. | n.a. |
| $PMA_{97}$-*b*-PSPA-$Na_{98}$‡ | 26.5 | 2.60 | 3.06 | 0.50 | 2.60 | 3.10 | 0.51 |

‡ extra DMSO *(i.e.* 400 eq to the initiator) was added into the BSPA monomer.

**Table S4:** Characteristics of the $PMA_x$-*b*-PSPA-$Na_y$ block copolymers produced through optimization of the one-pot polymerization.

| | $t_{MA}$ (h) | $t_{BSPA}$ (h) | conv.$_{MA}$‡ (%) | conv.$_{BSPA}$‡ (%) | $M_{n\ prot,\ NMR}$± (Da) | yield$_{prot}$ (mg) | yield$_{deprot}$ (mg) |
|---|---|---|---|---|---|---|---|
| $PMA_{113}$-*b*-PSPA-$Na_{72}$ | 2 | 16 | 98 | 72 | 27 900 | 173 | 364 |
| $PMA_{109}$-*b*-PSPA-$Na_{86}$ | 2 | 16 | 96 | 84 | 31 100 | 219 | 375 |
| $PMA_{97}$-*b*-PSPA-$Na_{98}$ | 2 | 16 | 97 | 98 | 33 000 | 238 | 411 |

*t* = time of reaction, ‡ determined by $^1$H NMR conversion samples; ± determined by $^1$H NMR from a combination of conversion samples and end-group analysis.

**Attempted polymerization of poly(3-isobutoxysulfopropyl acrylate)-*block*-poly(methyl acrylate) amphiphilic block copolymers (*i.e.,* reverse synthesis).**

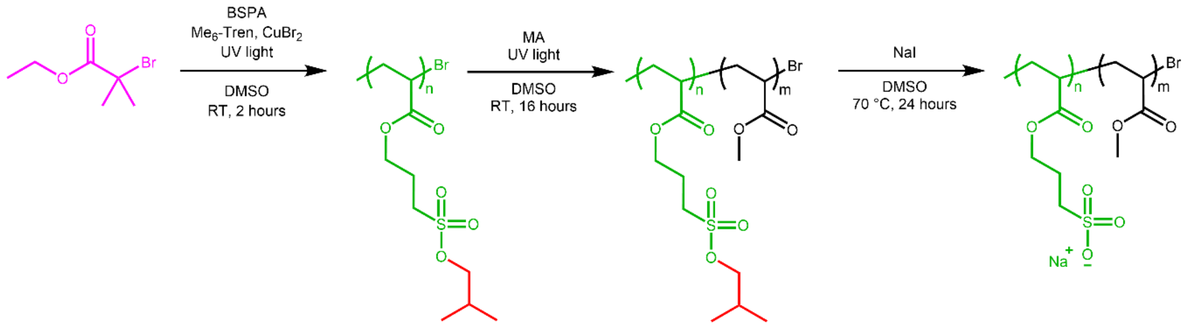


For the attempted reverse synthesis, EB*i*B (1 eq, 5.20 mg, 26.7 µmol, 155 µL of a stock solution of 103 mg EB*i*B in 3.07 mL DMSO), BSPA (98 eq, 650 mg, 2.60 mmol), $Me_6$-TREN (0.12 eq, 0.759 mg, 3.30 µmol) and $CuBr_2$ (0.02 eq, 0.121 mg, 0.543 µmol (130 µL of a stock solution of 29.6 mg $Me_6$-TREN and 4.72 mg $CuBr_2$ in 5.06 mL DMSO) and DMSO (400 eq, 469 µL, total volume of 738 µL) were charged into a Schlenk tube equipped with a stirring egg. The reaction mixture was deoxygenated via argon bubbling for 5 min and backfilled with argon before an aliquot was withdrawn under argon protection for $^1$H NMR sampling. The tube was then placed inside the light reactor and after 2 hours the light was switched off and aliquots for $^1$H NMR and SEC analyses (~ 2 drops each) were withdrawn under argon protection. In a separate vial, a solution of MA (112 eq, 257 mg, 2.99 mmol), 130 µL of the $Me_6$-TREN/$CuBr_2$ stock solution and DMSO (200 eq, 369 µL) was deoxygenated via 3 freeze-pump-thaw cycles, before being withdrawn and injected into the main Schlenk tube under argon protection. The solution was left to thoroughly mix for a few minutes before an aliquot for $^1$H NMR analysis (~ 2 drops) was withdrawn under argon protection. The light of the photoreactor was switched back on, and the reaction mixture was stirred for 16 hours, after which the light was switched off and the Schlenk tube was opened to air. Aliquots for $^1$H NMR and SEC analyses (~ 2 drops each) were withdrawn along with a larger aliquot (~ 25 drops each) that was purified by precipitation in cold 6:1 *n*-hexane:ethanol and dried *in vacuuo* for later analysis. In a third vial, NaI (3 eq to BSPA, 1.15 g, 7.69 mmol) was dissolved into 9 mL of DMSO before being added into the Schlenk tube. The vessel was then immersed in a pre-heated oil bath at 70 °C and stirred for 24 hours. The resulting dark brown mixture was precipitated in cold 1:2 *n*-hexane:ethanol, washed several times with 1:2 *n*-hexane:ethanol and one last time with pure *n*-hexane. The polymer was dried *in vacuuo* to yield a white powder. Yield: $PBSPA_{97}$-*b*-$PMA_{93}$: 105 mg, PSPA-$Na_{97}$-*b*-$PMA_{93}$: 556 mg. $^1$H NMR: $conv._{PBSPA}$ = 99 %, $conv._{PMA}$ = 95 %, $DP_{PBSPA}$ = 98, $DP_{PMA}$ = 93, $M_{n\ PBSPA\text{-}b\text{-}PMA}$ = 32 400 Da, $M_{n\ PSPA\text{-}Na\text{-}b\text{-}PMA}$ = 29 100 Da. SEC: $M_{n\ PBSPA}$ = 28 000 Da, Đ = 1.12, $M_{n\ PBSPA\text{-}b\text{-}PMA}$ = 41 600 Da, Đ = 1.16.

**One-pot synthesis of poly(methyl acrylate)-*block*-poly(3-isobutoxysulfopropyl acrylate) amphiphilic block copolymers.**

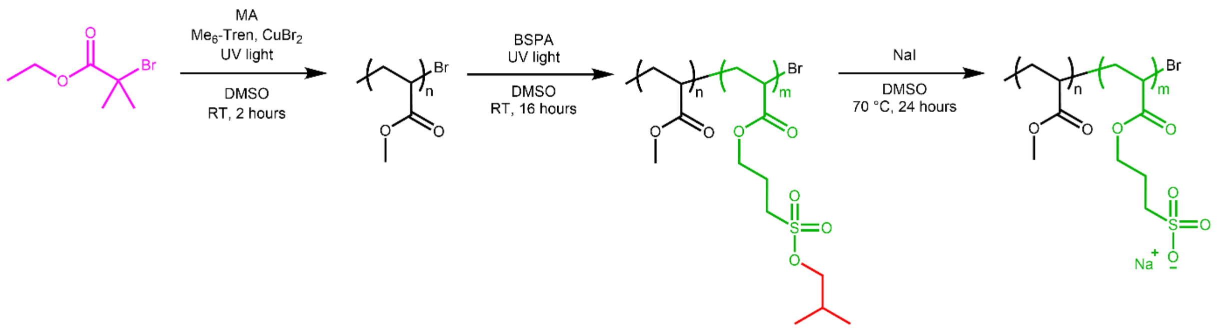


A typical example of one-pot polymerization can be found here. Detailed reaction mixture compositions of further reactions can be found in **Table S5**. EB*i*B (1 eq, 5.04 mg, 25.8 μmol, 138 μL of a stock solution of 109 mg EB*i*B in 3.02 mL DMSO), MA (116 eq, 258 mg, 3.00 mmol), $Me_6$-TREN (0.12 eq, 0.68 mg, 2.93 μmol) and $CuBr_2$ (0.02 eq, 0.12 mg, 0.54 μmol (45 μL of a stock solution of 31.8 mg $Me_6$-TREN and 4.45 mg $CuBr_2$ in 2.05 mL DMSO) and DMSO (200 eq, 186 μL, total volume of 369 μL) were charged into a Schlenk tube equipped with a stirring egg. The reaction mixture was deoxygenated via 3 freeze-pump-thaw cycles and backfilled with argon before an aliquot was withdrawn under argon protection for $^1$H NMR sampling. The tube was then placed inside the light reactor and after 2 hours the light was switched off and aliquots for $^1$H NMR and SEC analyses (~ 2 drops each) were withdrawn under argon protection. In a separate vial, a solution of BSPA (100 eq, 647 mg, 2.59 mmol) and DMSO (200 eq, 369 μL) was deoxygenated via argon bubbling for 5 min, before being withdrawn and injected into the main Schlenk tube under argon protection. The solution was left to thoroughly mix for a few minutes before an aliquot for $^1$H NMR analysis (~ 2 drops) was withdrawn under argon protection. The light of the photoreactor was switched back on, and the reaction mixture was stirred for 16 hours, after which the light was switched off and the Schlenk tube was opened to air. Aliquots for $^1$H NMR and SEC analyses (~ 2 drops each) were withdrawn along with a larger aliquot (~ 25 drops each) that was purified by precipitation in cold 6:1 *n*-hexane:ethanol and freeze-dried from 1,4-dioxane for later analysis. In a third vial, NaI (3 eq to BSPA, 1.26 g, 8.4 mmol) was dissolved into 9 mL of DMSO before being added into the Schlenk tube. The vessel was then immersed in a pre-heated oil bath at 70 °C and stirred for 24 hours. The resulting dark brown mixture was precipitated in cold 1:2 *n*-hexane:ethanol, washed several times with 1:2 *n*-hexane:ethanol and one last time with pure *n*-hexane. The polymer was redissolved in a minimal volume of 1:1 1,4-dioxane:water mixture and freeze-dried overnight to yield a white powder. See **Table S6** for the characterization of the blocks.

**Table S5:** Compositions of the reaction mixtures used for the synthesis of $PMA_x$-*b*-PSPA-$Na_y$ block copolymers in one-pot.

| | block 1 | | | | block 2 | | |
|---|---|---|---|---|---|---|---|
| | EB*i*B (μmol) | MA (mmol) | $Me_6$-TREN (μmol) | $CuBr_2$ (μmol) | BSPA (mmol) | $Me_6$-TREN (μmol) | $CuBr_2$ (μmol) |
| $PMA_{243}$-*b*-PSPA-$Na_{48}$ | 26.5 | 6.45 | 3.10 | 0.52 | 1.30 | 2.77 | 0.46 |
| $PMA_{193}$-*b*-PSPA-$Na_{94}$ | 25.8 | 5.19 | 3.14 | 0.53 | 2.57 | 3.14 | 0.53 |
| $PMA_{146}$-*b*-PSPA-$Na_{164}$ | 26.2 | 4.01 | 3.16 | 0.53 | 4.08 | 3.16 | 0.53 |
| $PMA_{98}$-*b*-PSPA-$Na_{228}$ | 26.0 | 2.66 | 3.16 | 0.52 | 5.15 | 3.16 | 0.52 |
| $PMA_{47}$-*b*-PSPA-$Na_{254}$ | 26.1 | 1.30 | 3.16 | 0.52 | 6.44 | 3.22 | 0.53 |

**Table S6:** Characteristics of the various $PMA_x$-*b*-PSPA-$Na_y$ block copolymers obtained through one-pot polymerization.

| | $t_{MA}$ (h) | $t_{BSPA}$ (h) | conv.$_{MA}$‡ (%) | conv.$_{BSPA}$‡ (%) | $M_{n\ prot,\ NMR}$± (Da) | yield$_{prot}$ (mg) | yield$_{deprot}$ (mg) |
|---|---|---|---|---|---|---|---|
| $PMA_{231}$-*b*-PSPA-$Na_{48}$ | 2 | 16 | 95 | 99 | 32 100 | 139 | 572 |
| $PMA_{193}$-*b*-PSPA-$Na_{94}$ | 2 | 18 | 96 | 97 | 40 300 | 175 | 660 |
| $PMA_{146}$-*b*-PSPA-$Na_{164}$ | 2 | 16 | 96 | 99 | 53 800 | 234 | 880 |
| $PMA_{98}$-*b*-PSPA-$Na_{228}$ | 2 | 16 | 95 | 97 | 65 600 | 118 | 1401 |
| $PMA_{47}$-*b*-PSPA-$Na_{254}$ | 2 | 16 | 95 | 95 | 67 000 | 130 | 966 |

*t* = time of reaction, ‡ determined by $^1H$ NMR conversion samples; ± determined by $^1H$ NMR from a combination of conversion samples and end-group analysis.

**Synthesis of multiple poly(methyl acrylate)-*block*-poly(sulfopropyl acrylate) amphiphilic block copolymers through sampling the one-pot polymerization.**

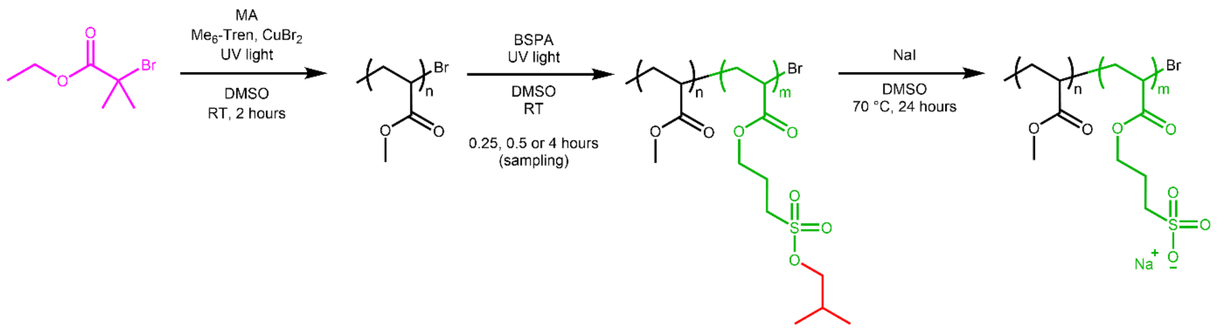


EB*i*B (1 eq, 5.11 mg, 26.2 µmol, 153 µL of a stock solution of 103 mg EB*i*B in 3.07 mL DMSO), MA (105 eq, 237 mg, 2.76 mmol), $Me_6$-TREN (0.12 eq, 0.719 mg, 3.13 µmol) and $CuBr_2$ (0.02 eq, 0.116 mg, 0.520 µmol, both from 141 µL of a stock solution of 26.0 mg $Me_6$-TREN and 4.18 mg $CuBr_2$ in 5.60 mL DMSO) and DMSO (400 eq, 449 µL, total volume of 738 µL) were charged into a Schlenk tube equipped with a stirring egg. The reaction mixture was deoxygenated via 3 freeze-pump-thaw cycles and backfilled with argon before an aliquot was withdrawn under argon protection for $^1$H NMR sampling. The tube was then placed inside the light reactor and after 2.5 hours, the light was switched off and aliquots for $^1$H NMR and SEC analyses (~ 2 drops each) were withdrawn under argon protection. In a separate vial, a solution of BSPA (197 eq, 1,29 g, 5.16 mmol), $Me_6$-TREN (0.12 eq, 0.749 mg, 3.26 µmol) and $CuBr_2$ (0.02 eq, 0.121 mg, 0.543 µmol (147 µL of the stock solution) and DMSO (200 eq, 598 µL, total volume of 738 µL) was deoxygenated via argon bubbling for 5 min, before being injected into the main Schlenk tube under argon protection. The solution was left to thoroughly mix for a few minutes before an aliquot for $^1$H NMR analysis (~ 2 drops) was withdrawn under argon protection. The light of the photoreactor was switched back on and the reaction mixture was stirred. After 15 min, the light was switched off to sample diblock 1: aliquots for $^1$H NMR and SEC analyses (~ 2 drops each) were withdrawn, alongside one for purified $^1$H NMR (~ 5 drops) and a larger one for deprotection (~ 750 µL). The reaction was then allowed to proceed further, and diblock 2 and diblock 3 were similarly withdrawn after 30 min and 4 hours respectively. The three large aliquots were then diluted with 6 mL of DMSO and NaI (770 mg, 5.13 mmol) was introduced. These solutions were deprotected at 70 °C for 20 hours, before precipitation in cold 1:2 *n*-hexane:ethanol, washing once with 1:2 *n*-hexane:ethanol and once with pure *n*-hexane before drying under high vacuum. See **Table S7** for the characterization of the blocks.

**Table S7:** Characteristics of the three $PMA_{102}$-*b*-PSPA-$Na_x$ block copolymers obtained by sampling the one-pot polymerization.

| | MA | | BSPA | | NMR | | | SEC | |
|---|---|---|---|---|---|---|---|---|---|
| | *t* (h) | conv.‡ (%) | *t* (h) | conv.‡ (%) | $x_{SPA}$± (mol.%) | $M_{n\ prot.}$± (Da) | $M_{n\ deprot.}$± (Da) | $M_{n\ prot}$* (Da) | Đ* |
| $PMA_{102}$ | 2.5 | 97 | n.a. | n.a. | n.a. | 9 000 | n.a. | 11 400 | 1.10 |
| $PMA_{102}$-*b*-PSPA-$Na_{93}$ | n.a. | n.a. | 0.25 | 41 | 48 | 32 200 | 29 000 | 39 100 | 1.10 |
| $PMA_{102}$-*b*-PSPA-$Na_{140}$ | n.a. | n.a. | 0.5 | 63 | 58 | 44 000 | 39 200 | 48 300 | 1.10 |
| $PMA_{102}$-*b*-PSPA-$Na_{207}$ | n.a. | n.a. | 4 | 98 | 67 | 60 700 | 53 700 | 65 200 | 1.14 |

*t* = time of reaction, ‡ determined by $^1$H NMR conversion samples; ± determined by $^1$H NMR from a combination of conversion samples and end-group analysis; * determined by SEC in DMF with 0.01 M LiBr on the purified protected intermediate and calibrated against near-monodisperse PMMA standards.

# Supplementary Figures

**S1: Photographs.**

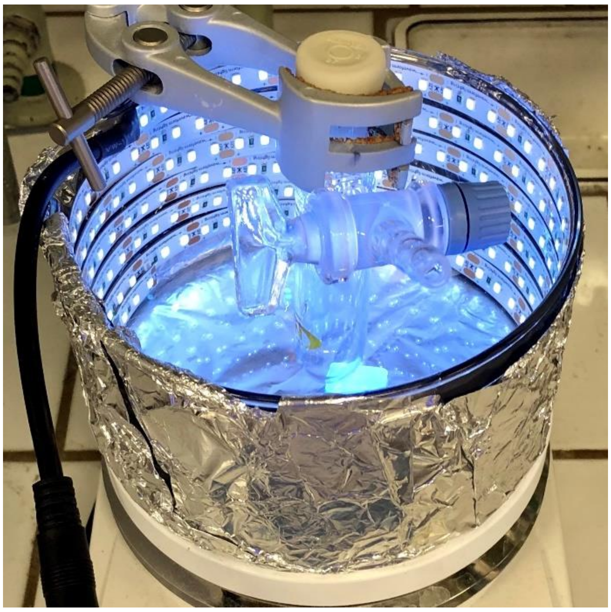

**Figure S1-1:** Photograph of the light reactor used in this study (λ = 365 nm, ~ 29 W output).

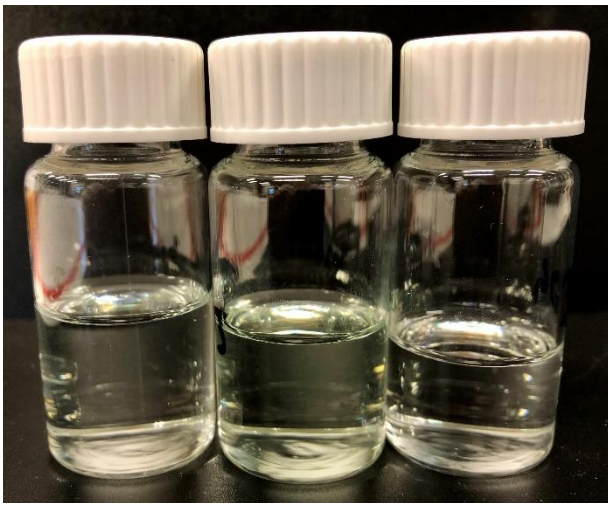

**Figure S1-2:** Photograph of various reaction mixtures after copper-catalyzed radical polymerization: (left) copper(0)-mediated radical polymerization after purification, (middle) copper(0)-mediated radical polymerization before purification and photo-RDRP used in this study (right).

**S2: Photo-RDRP of homopolymers.**

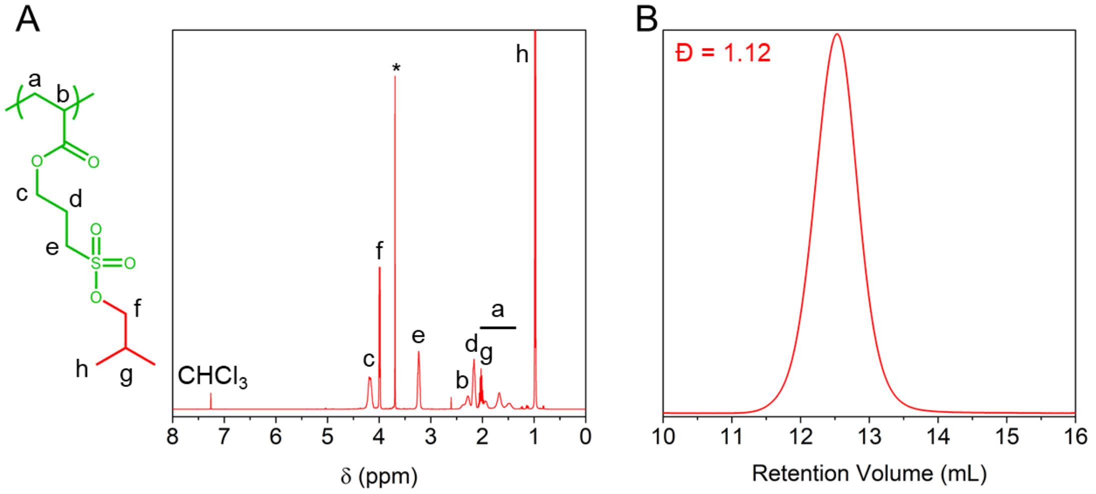


**Figure S2-1:** Characterization of a purified $PBSPA_{99}$ homopolymer produced via photo-RDRP: (A) $^1H$ NMR (400 MHz, $CDCl_3$) and (B) SEC measured in DMF with 0.01 M LiBr. *: residual 1,4-dioxane.

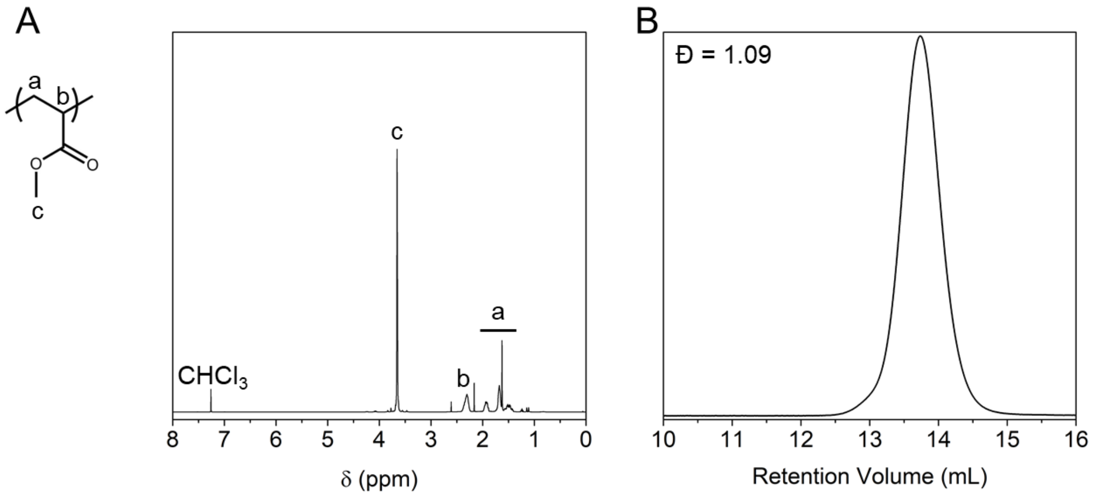


**Figure S2-2:** Characterization of a purified $PMA_{97}$ homopolymer produced via photo-RDRP: (A) $^1H$ NMR (400 MHz, $CDCl_3$) and (B) SEC measured in DMF with 0.01 M LiBr.

**S3: Deprotection of isobutoxy-protected homopolymer.**

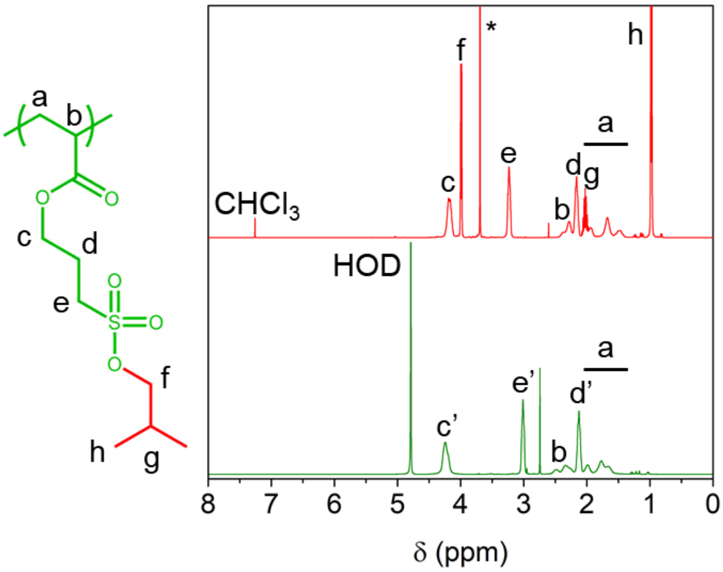


**Figure S3:** $^1$H NMR spectra (400 MHz) of a protected PBSPA$_{99}$ homopolymer (red, $CDCl_3$) and its nucleophile-deprotected analogue PSPA-Na$_{99}$ (green, $D_2O$).

The complete disappearance of signals f ($CH_2$, 2H, 4.0 ppm), g (CH, 1H, 2.0 ppm) and h ($CH_3$, 6H, 1.0 ppm) from the isobutoxy protective groups is evidenced and confirms the quantitative deprotection, yielding a PSPA-Na$_{99}$ fully anionic homopolymer.

## S4: Kinetics of photo-RDRP homopolymerizations.

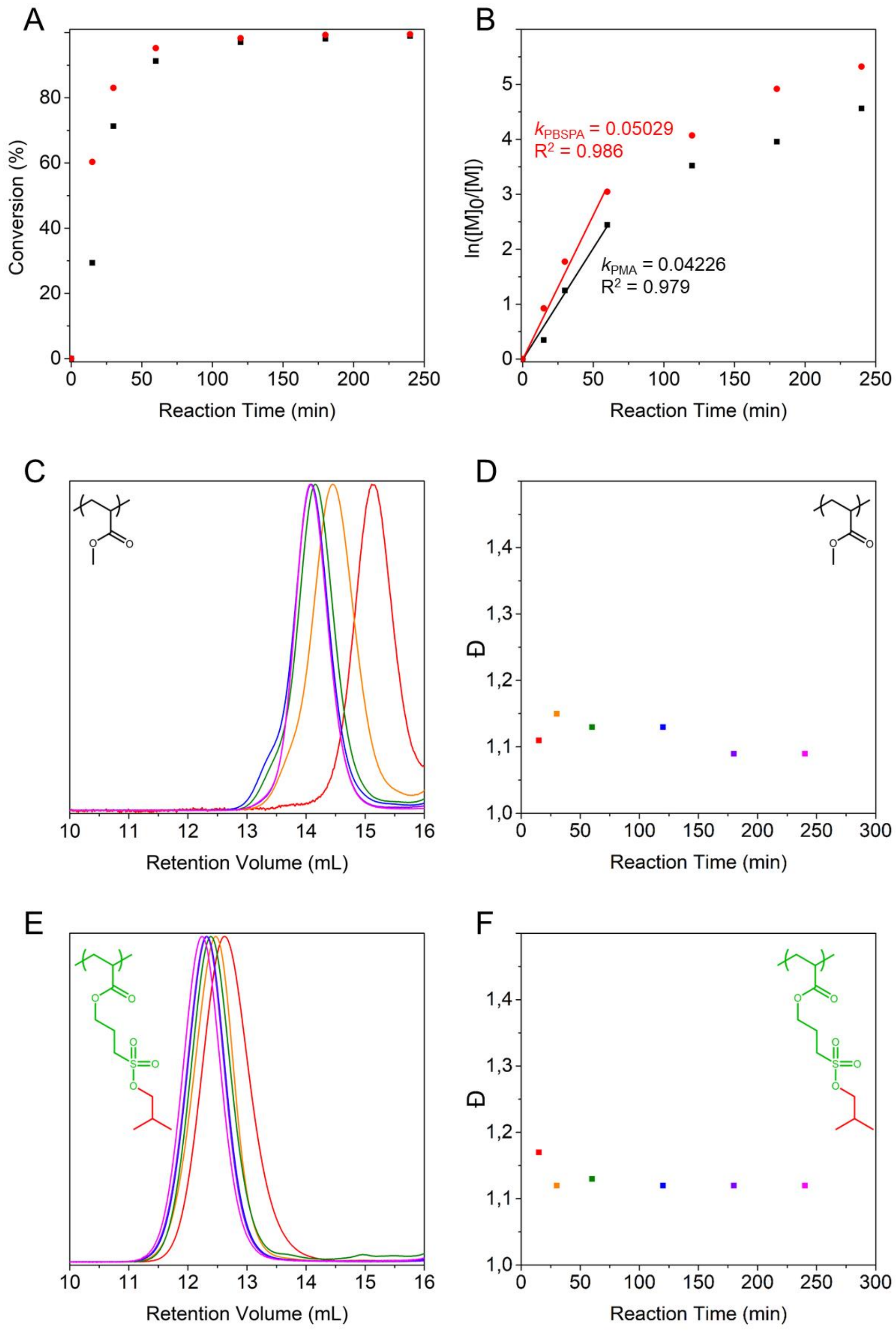


**Figure S4:** Kinetics of photo-RDRP of PMA and PBSPA homopolymers. Comparative plots of (A) conversion and (B) ln([M]$_0$/[M]) as a function of time for PMA (black) and PBSPA (red). Corresponding (C,E) SEC elugrams and (D, F) dispersity values measured in DMF with 0.01 M LiBr.

**S5: Chain extension tests on PMA and PBSPA homopolymers.**

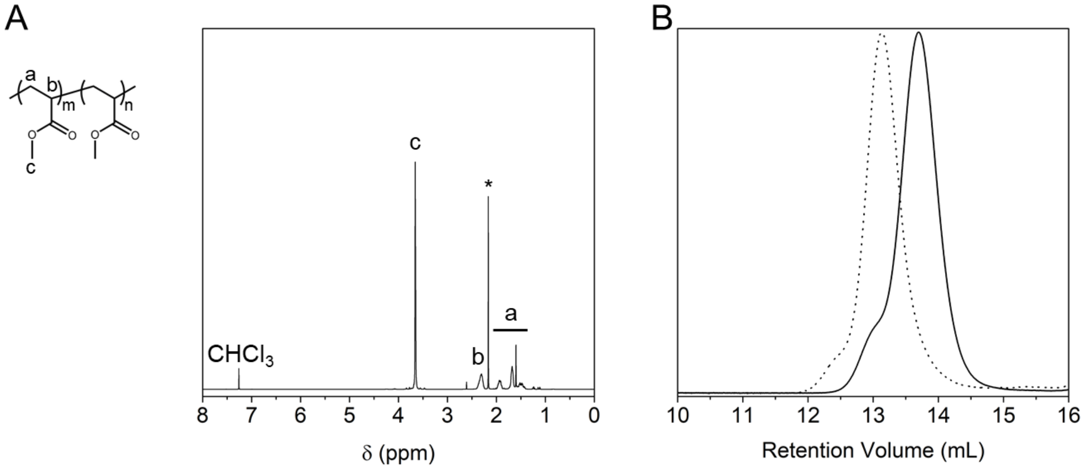


**Figure S5-1:** Homopolymerization chain extension test of PMA. (A) $^{1}H$ NMR spectra (400 MHz, $CDCl_3$) of $PMA_{99}$-*b*-$PMA_{87}$ and (B) SEC elugrams of $PMA_{99}$ (solid line) and chain extended $PMA_{99}$-*b*-$PMA_{87}$ (dotted line) measured in DMF with 0.01 M LiBr. *: residual acetone.

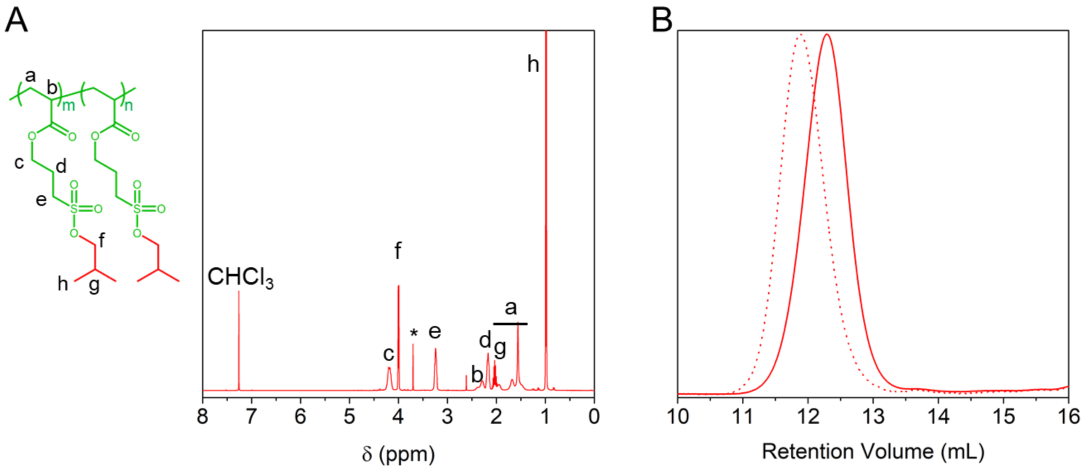


**Figure S5-2:** Homopolymerization chain extension test of PBSPA. (A) $^{1}H$ NMR spectra (400 MHz, $CDCl_3$) of $PBSPA_{103}$-*b*-$PBSPA_{70}$ and (B) SEC elugrams of $PBSPA_{103}$ (solid line) and chain extended $PMA_{103}$-*b*-$PBSPA_{70}$ (dotted line) measured in DMF with 0.01 M LiBr. *: residual 1,4-dioxane.

**S6: Optimization of the one-pot polymerization of $PMA_x$-b-PSPA-$Na_y$.**

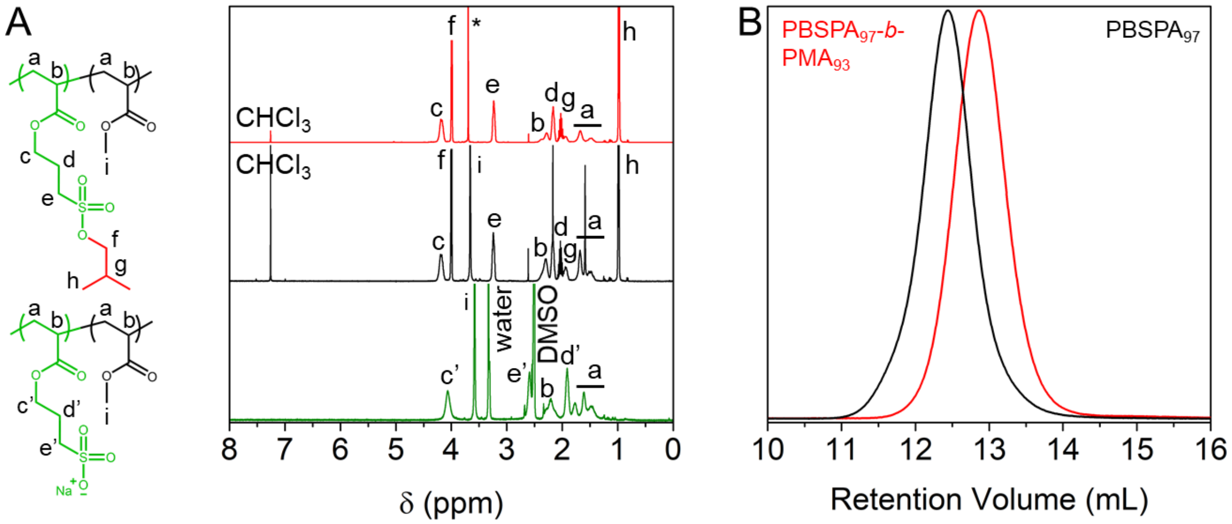


**Figure S6-1:** Attempt to produce a PSPA-Na-*b*-PMA block copolymer (*i.e.,* reverse synthesis strategy). (A) $^{1}$H NMR spectra (400 MHz) of a PBSPA (red, $CDCl_3$), $PBSPA_{97}$-*b*-$PMA_{93}$ (black, $CDCl_3$) and PSPA-$Na_{97}$-*b*-$PMA_{93}$ (green, DMSO-$d_6$. Corresponding SEC elugrams of the $PBSPA_{97}$ (first block, red) and $PBSPA_{97}$-b-$PMA_{93}$ (protected diblock, black) measured in DMF with 0.01 M LiBr.*: residual 1,4-dioxane.

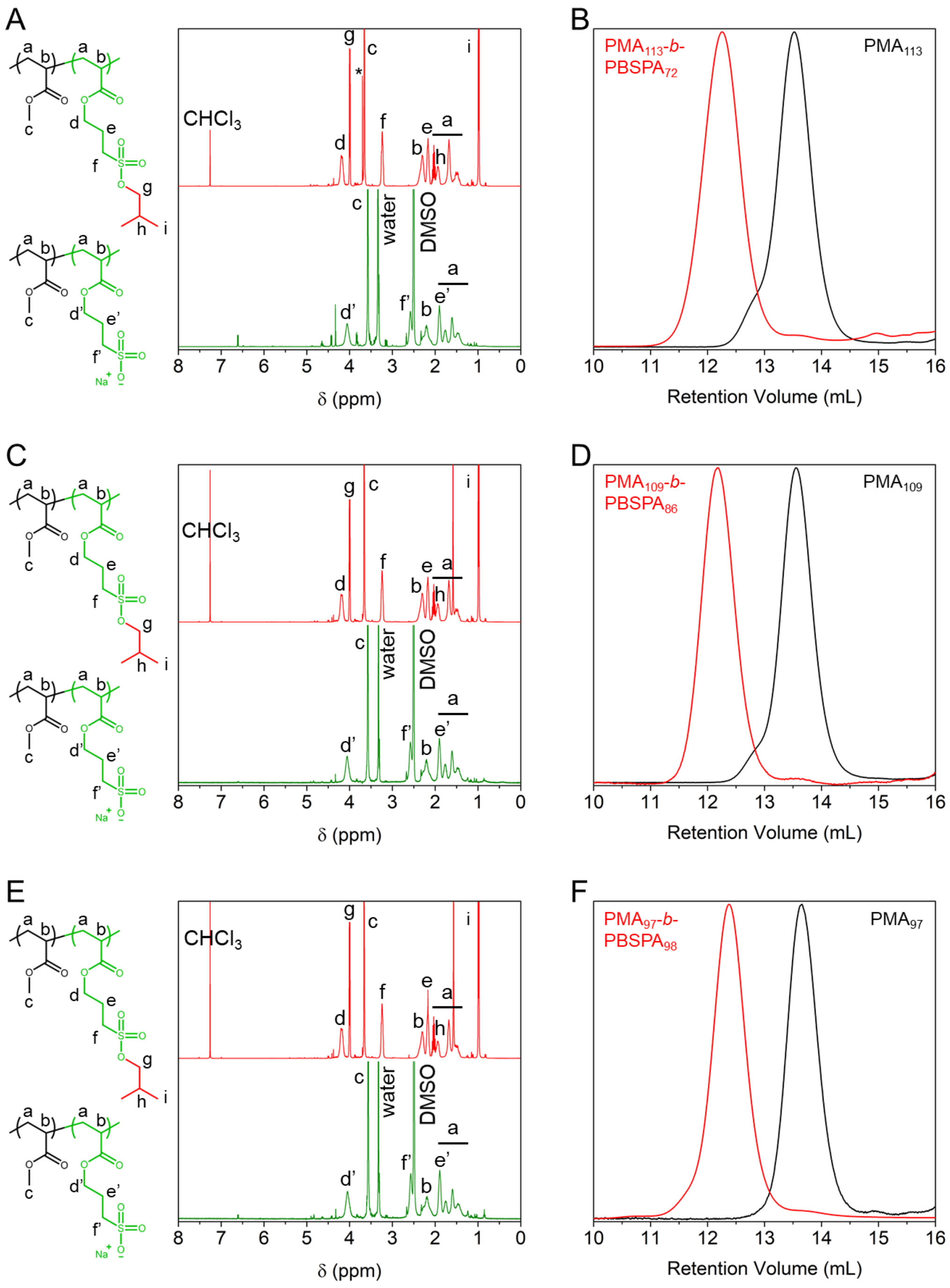


**Figure S6-2:** Optimization of the *in-situ* chain extension of PMA with BSPA monomer. $^{1}$H NMR spectra (400 MHz) of the PMA$_x$-*b*-PBSPA$_y$ ($CDCl_3$) and PMA$_x$-*b*-PSPA-Na$_y$ (DMSO-$d_6$) block copolymers obtained through (A) BSPA, (C) {BSPA+DMSO} and (E) {BSPA+DMSO+Me$_6$-TREN+$CuBr_2$} addition under inert gas protection. (B,D,F) Corresponding SEC elugrams of the PMA$_x$ first block (black) and chain extended PMA$_x$-*b*-PBSPA$_y$ (red) measured in DMF with 0.01 M LiBr. *: residual 1,4-dioxane.

**S7: DLS of nanoparticles of the optimized block copolymer.**

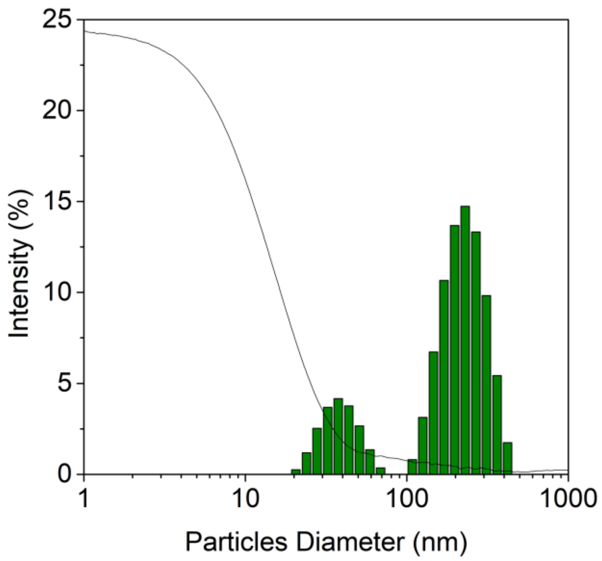


**Figure S7:** DLS intensity plot (bars) and corresponding correlogram function (solid lines) of the $PMA_{97}$-*b*-PSPA-$Na_{98}$ amphiphilic block copolymer produced through optimized one-pot polymerization. Here, $D_h$ = 185.6 ± 12.0 nm and ζ = 35.4 ± 2.4 mV.

**S8: Electron microscopy images of nanoparticles of the optimized block copolymer.**

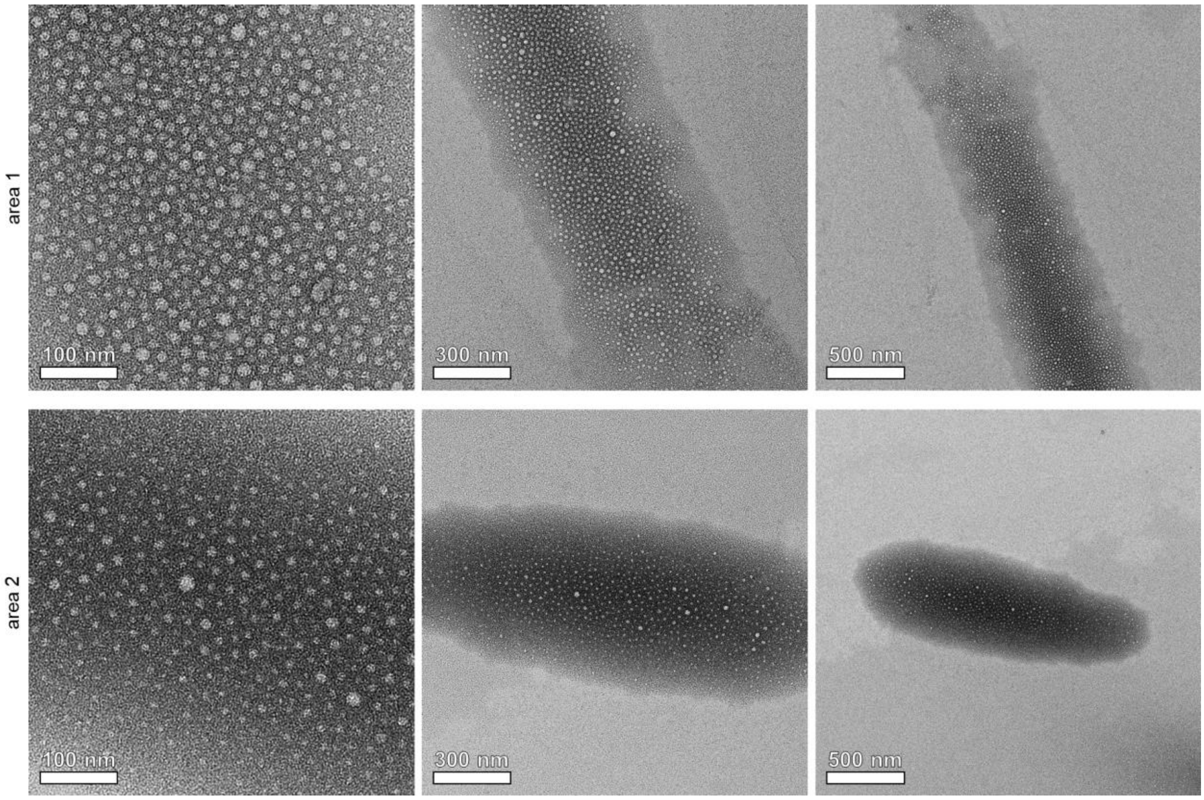


**Figure S8:** TEM images of uranyl acetate-stained nanoparticles self-assembled from a solution of $PMA_{97}$-b-PSPA-$Na_{98}$ amphiphilic block copolymer at 1 g $L^{-1}$ in 10 mM $KNO_3$.

## S9: $^{1}$H NMR and SEC analyses of the $PMA_x$-*b*-$PSPA\text{-}Na_y$ block copolymers.

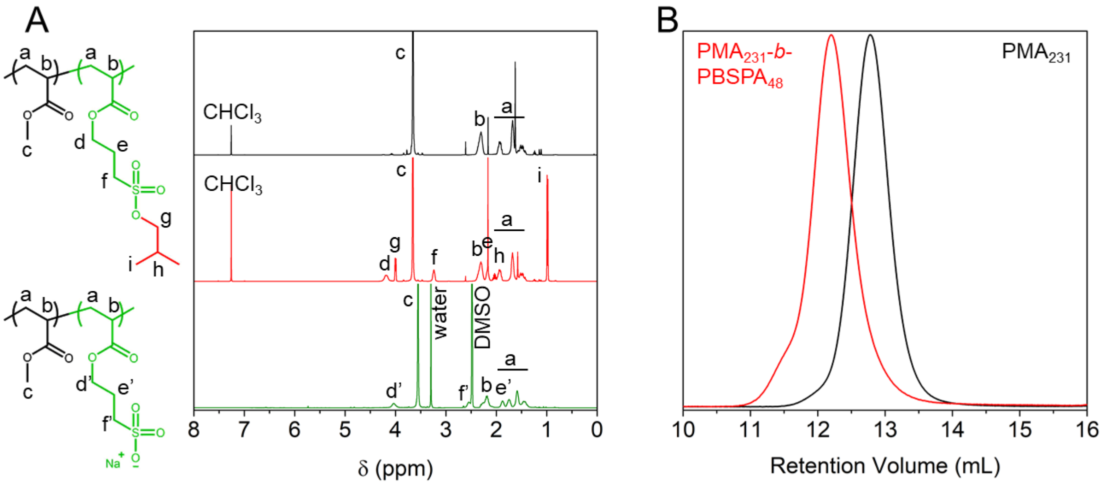


**Figure S9-1:** Analysis of the polymers involved in the one-pot synthesis of $PMA_{231}$-*b*-$PSPA\text{-}Na_{48}$. (A) $^{1}$H NMR spectra (400 MHz) of a PMA ($CDCl_3$, black), $PMA_{231}$-*b*-$PBSPA_{48}$ ($CDCl_3$, red) and $PMA_{231}$-*b*-$PSPA\text{-}Na_{48}$ (DMSO-*d*$_6$, green). (B) SEC elugrams of $PMA_{231}$ (black) and $PMA_{231}$-*b*-$PBSPA_{48}$ (red) measured at 50 °C in DMF with 0.01 M LiBr.

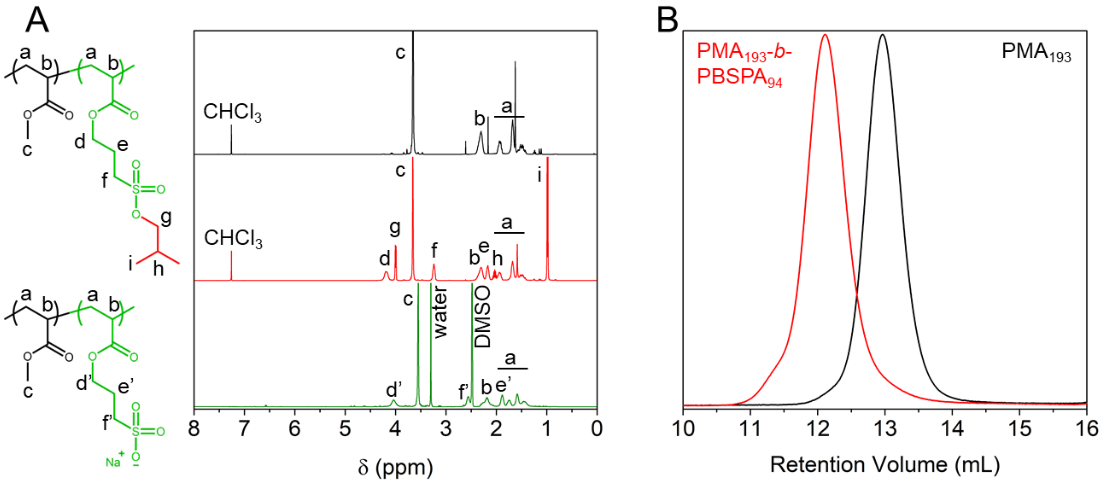


**Figure S9-2:** Analysis of the polymers involved in the one-pot synthesis of $PMA_{193}$-*b*-$PSPA\text{-}Na_{94}$. (A) $^{1}$H NMR spectra (400 MHz) of a PMA ($CDCl_3$, black), $PMA_{193}$-*b*-$PBSPA_{94}$ ($CDCl_3$, red) and $PMA_{193}$-*b*-$PSPA\text{-}Na_{94}$ (DMSO-*d*$_6$, green). (B) SEC elugrams of $PMA_{193}$ (black) and $PMA_{193}$-*b*-$PBSPA_{94}$ (red) measured at 50 °C in DMF with 0.01 M LiBr.

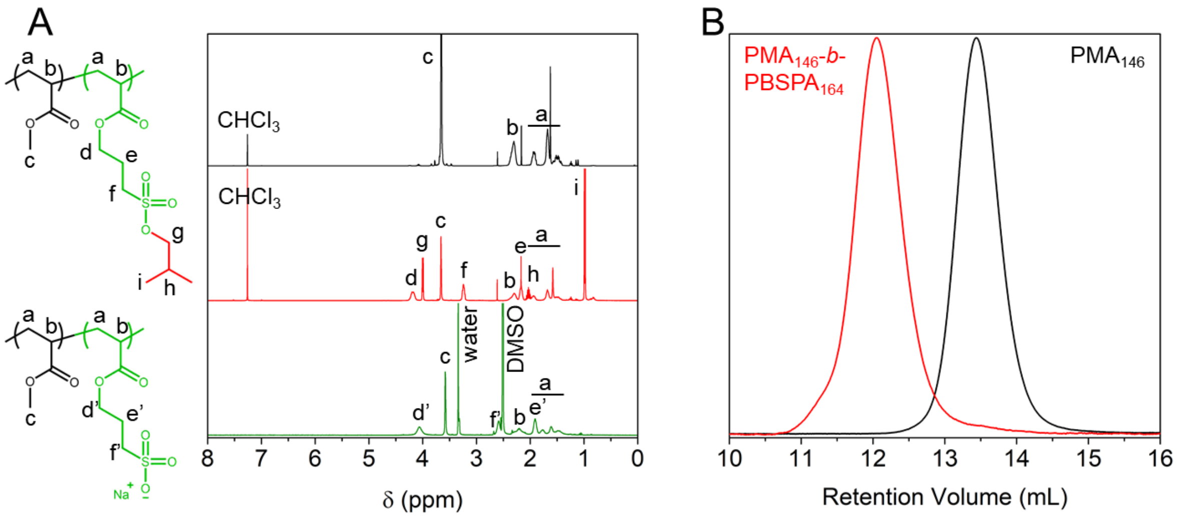


**Figure S9-3:** Analysis of the polymers involved in the one-pot synthesis of PMA$_{146}$-*b*-PSPA-Na$_{164}$. (A) $^1$H NMR spectra (400 MHz) of a PMA ($CDCl_3$, black), PMA$_{146}$-*b*-PBSP164$_8$ ($CDCl_3$, red) and PMA$_{146}$-*b*-PSPA-Na$_{164}$ (DMSO-$d_6$, green). (B) SEC elugrams of PMA$_{146}$ (black) and PMA$_{146}$-*b*-PBSPA$_{164}$ (red) measured at 50 °C in DMF with 0.01 M LiBr.

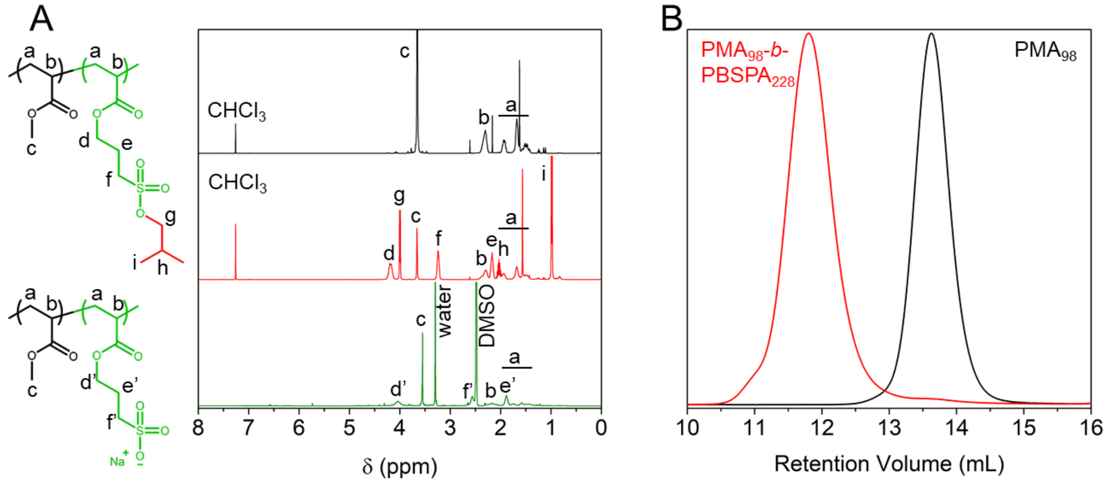


**Figure S9-4:** Analysis of the polymers involved in the one-pot synthesis of PMA$_{98}$-*b*-PSPA-Na$_{228}$. (A) $^1$H NMR spectra (400 MHz) of a PMA ($CDCl_3$, black), PMA$_{98}$-*b*-PBSPA$_{228}$ ($CDCl_3$, red) and PMA$_{98}$-*b*-PSPA-Na$_{228}$ (DMSO-$d_6$, green). (B) SEC elugrams of PMA$_{98}$ (black) and PMA$_{98}$-*b*-PBSPA$_{228}$ (red) measured at 50 °C in DMF with 0.01 M LiBr.

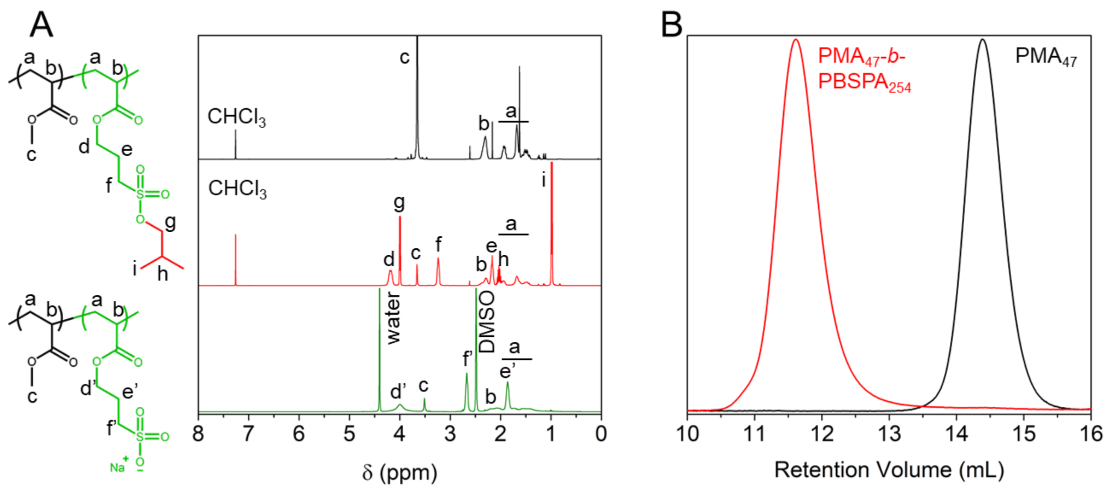


**Figure S9-5:** Analysis of the polymers involved in the one-pot synthesis of $PMA_{47}$-*b*-$PSPA$-$Na_{254}$. (A) $^1H$ NMR spectra (400 MHz) of a PMA ($CDCl_3$, black), $PMA_{47}$-*b*-$PBSPA_{254}$ ($CDCl_3$, red) and $PMA_{47}$-*b*-$PSPA$-$Na_{254}$ (50:50 DMSO-$d_6$:$D_2O$, green). (B) SEC elugrams of $PMA_{47}$ (black) and $PMA_{47}$-*b*-$PBSPA_{254}$ (red) measured at 50 °C in DMF with 0.01 M LiBr.

## S10: Infrared spectroscopy of homopolymers and block copolymers.

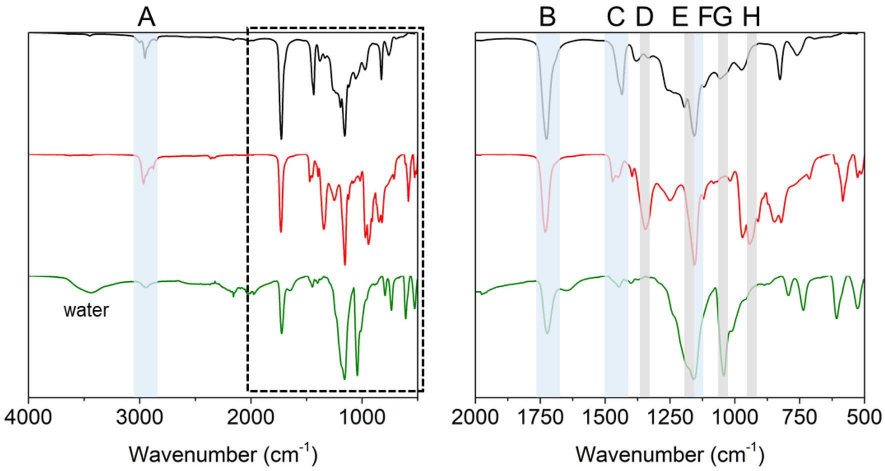


**Figure S10-1:** Infrared spectra of various homopolymers: $PMA_{97}$ (black), $PBSPA_{99}$ (red) and PSPA-$Na_{99}$ (green). Assigned signals are: (A) -CH stretching backbone (2954 $cm^{-1}$), (B) -CH bending backbone (1436 $cm^{-1}$), (C) -C=O acrylate (1726 $cm^{-1}$), (D) S=O sulfonate (1347 $cm^{-1}$), (E) S=O sulfonate (1192 $cm^{-1}$), (F) -C-O acrylate (1156 $cm^{-1}$), (G) S=O sulfonate (1045 $cm^{-1}$) and (H) S-O-R sulfonic ester (941 $cm^{-1}$).

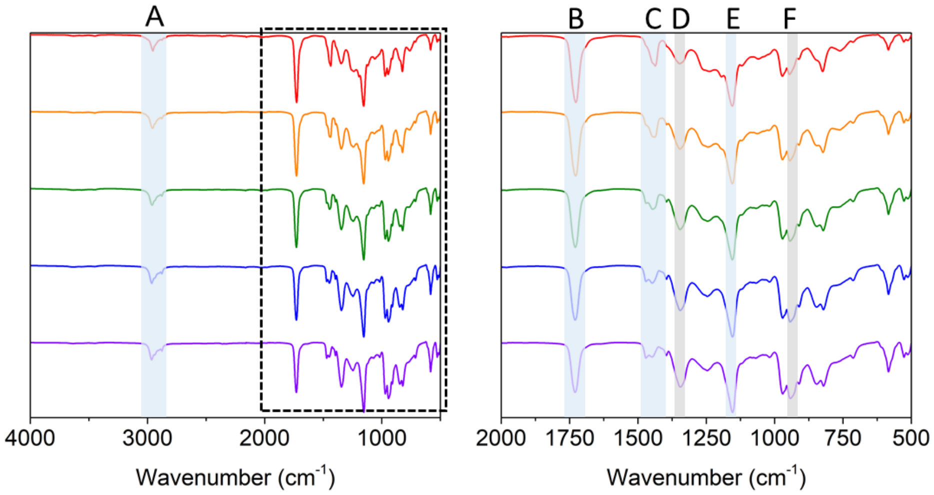


**Figure S10-2:** Infrared spectra of the protected block copolymers: $PMA_{231}$-*b*-$PBSPA_{48}$ ($x_{BSPA}$ = 0.17, red), $PMA_{193}$-*b*-$PBSPA_{94}$ ($x_{BSPA}$ = 0.33, orange), $PMA_{146}$-*b*-$PBSPA_{164}$ ($x_{BSPA}$ = 0.53, green), $PMA_{98}$-*b*-$PBSPA_{228}$ ($x_{BSPA}$ = 0.70, blue) and $PMA_{47}$-*b*-$PBSPA_{254}$ ($x_{BSPA}$ = 0.84, violet). Assigned signals are: (A) -CH stretching backbone (2954 $cm^{-1}$), (B) -CH bending backbone (1436 $cm^{-1}$), (C) -C=O acrylate (1726 $cm^{-1}$), (D) S=O sulfonate (1347 $cm^{-1}$), (E) -C-O acrylate (1156 $cm^{-1}$) and (F) S-O-R sulfonic ester (941 $cm^{-1}$).

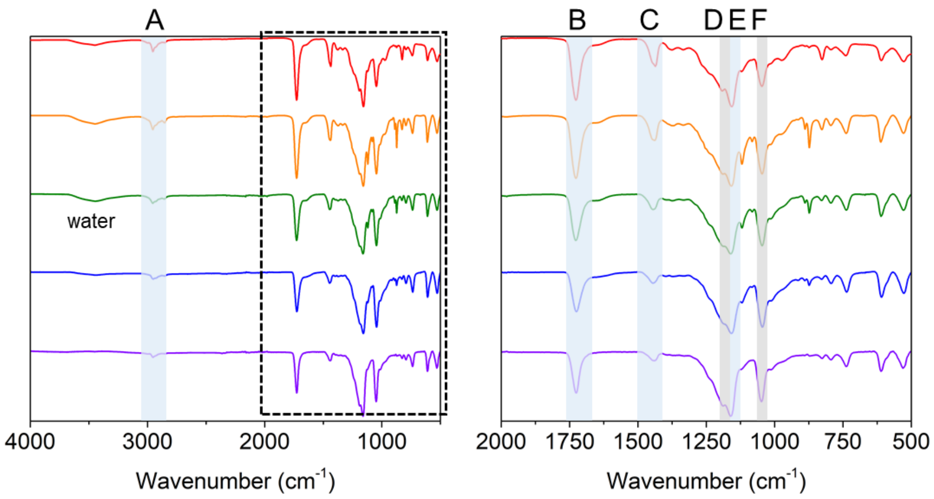


**Figure S10-3:** Infrared spectra of the deprotected amphiphilic block copolymers: $PMA_{231}$-*b*-$PSPA\text{-}Na_{48}$ ($x_{SPA\text{-}Na}$ = 0.17, red), $PMA_{193}$-*b*-$PSPA\text{-}Na_{94}$ ($x_{SPA\text{-}Na}$ = 0.33, orange), $PMA_{146}$-*b*-$PSPA\text{-}Na_{164}$ ($x_{SPA\text{-}Na}$ = 0.53, green), $PMA_{98}$-*b*-$PSPA\text{-}Na_{228}$ ($x_{SPA\text{-}Na}$ = 0.70, blue) and $PMA_{47}$-*b*-$PSPA\text{-}Na_{254}$ ($x_{SPA\text{-}Na}$ = 0.84, violet). Assigned signals are: (A) -CH stretching backbone (2954 $cm^{-1}$), (B) -CH bending backbone (1436 $cm^{-1}$), (C) -C=O acrylate (1726 $cm^{-1}$), (D) S=O sulfonate (1192 $cm^{-1}$), (E) -C-O acrylate (1156 $cm^{-1}$) and (F) S=O sulfonate (1045 $cm^{-1}$).

**S11: Thermal analyses of homo- and block copolymers.**

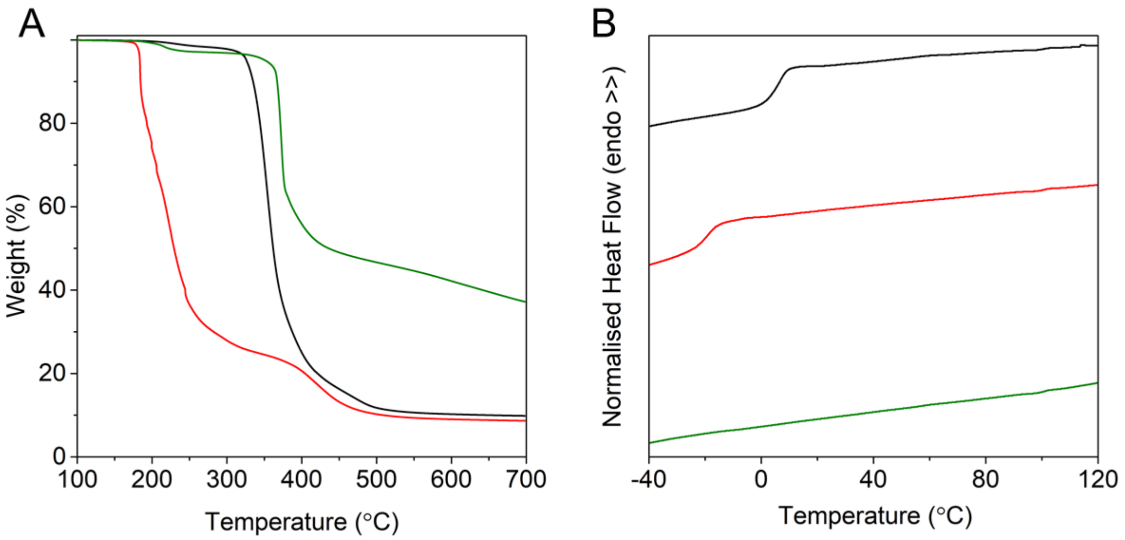


**Figure S11:** (A) Thermogravimetric and (B) differential scanning calorimetry analyses of various homopolymers: $PMA_{97}$ (black), $PBSPA_{99}$ (red) and PSPA-$Na_{99}$ (green).

**Table S8:** Degradation and glass transition temperatures of various homopolymers.

| | $T_{deg\ 1}$ ‡ (°C) | $W_{deg\ 1}$ ‡ (%) | $T_{deg\ 2}$ ‡ (°C) | $W_{deg\ 2}$ ‡ (%) | $T_g$ ± (°C) |
|---|---|---|---|---|---|
| $PMA_{97}$ | 352 | 83.5 | 467 | 6.1 | 5.2 |
| $PBSPA_{99}$ | 194+214 | 75.1 | 421 | 16.1 | -19.6 |
| PSPA-$Na_{99}$ | 216 | 2.7 | 374 | 58.7 | n.a. |

‡ determined by TGA at a rate of 10 °C $min^{-1}$ and temperature corresponding to the maximal rate at $T_{deg}$ (*i.e*. decomposition temperature), ± determined by DSC on the second heating ramp at a rate of 10 °C $min^{-1}$.

**Table S9:** Degradation and glass transition temperatures of the various $PMA_x$-*b*-$PBSPA_y$ protected block copolymers.

| | $x_{BSPA-Na}$ ‡ (mol.%) | $T_{deg\,1}$ ‡ (°C) | $W_{deg\,1}$ ‡ (%) | $T_{deg\,2}$ ‡ (°C) | $W_{deg\,2}$ ‡ (%) | $T_g$ ± (°C) | |
|---|---|---|---|---|---|---|---|
| $PMA_{231}$-*b*-$PBSPA_{48}$ | 17 | 200+247 | 11.7+17.1 | 391 | 65.4 | -7.5 | 9.8 |
| $PMA_{193}$-*b*-$PBSPA_{94}$ | 33 | 193+235 | 20.0+23.9 | 383 | 55.4 | -5.7 | 12.4 |
| $PMA_{146}$-*b*-$PBSPA_{164}$ | 50 | 199 | 63.6 | 389 | 33.3 | -9.3 | 10.8 |
| $PMA_{98}$-*b*-$PBSPA_{228}$ | 70 | 200 | 68.1 | 394 | 29.2 | -7.8 | n.a. |
| $PMA_{47}$-*b*-$PBSPA_{254}$ | 84 | 198 | 73.5 | 413 | 21.5 | -9.8 | n.a. |

‡ determined by $^1$H NMR from a combination of conversion and end-group analysis, ‡ determined by TGA at a rate 10 °C $min^{-1}$ and temperature corresponding to the maximal rate at $T_{deg}$ (*i.e*. decomposition temperature), ± determined by DSC on the second heating ramp at a rate of 10 °C $min^{-1}$.

**Table S10:** Degradation and glass transition temperatures of the various $PMA_x$-*b*-PSPA-$Na_y$ amphiphilic block copolymers.

| | $x_{BSPA-Na}$ ‡ (mol.%) | $T_{deg\,1}$ ‡ (°C) | $W_{deg\,1}$ ‡ (%) | $T_{deg\,2}$ ‡ (°C) | $W_{deg\,2}$ ‡ (%) | $W_{char}$ ‡ (%) | $T_g$ ± (°C) |
|---|---|---|---|---|---|---|---|
| $PMA_{231}$-*b*-PSPA-$Na_{48}$ | 17 | 381 | 70.5 | 475 | 7.3 | 19.3 | 16.9 |
| $PMA_{193}$-*b*-PSPA-$Na_{94}$ | 33 | 378 | 60.6 | 467 | 10.1 | 27.9 | n.a. |
| $PMA_{146}$-*b*-PSPA-$Na_{164}$ | 50 | 376 | 62.5 | n.a. | n.a. | 30.2 | n.a. |
| $PMA_{98}$-*b*-PSPA-$Na_{228}$ | 70 | 379 | 69.4 | n.a. | n.a. | 36.7 | n.a. |
| $PMA_{47}$-*b*-PSPA-$Na_{254}$ | 84 | 368 | 42.3 | 415 | 16.4 | 40.4 | n.a. |

‡ determined by $^1$H NMR from a combination of conversion and end-group analysis, ‡ determined by TGA at a rate 10 °C $min^{-1}$ and temperature corresponding to the maximal rate at $T_{deg}$ (*i.e*. decomposition temperature), ± determined by DSC on the second heating ramp at a rate of 10 °C $min^{-1}$.

**S12: Electron microscopy images of the $PMA_x$-*b*-PSPA-$Na_y$-based nanoparticles.**

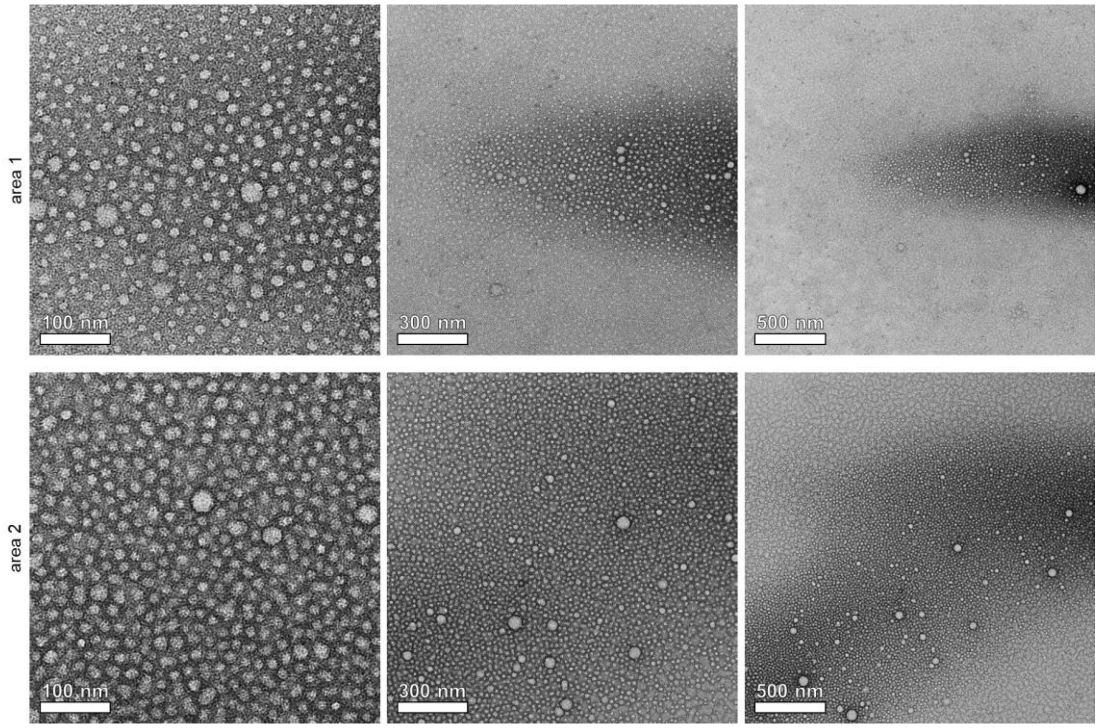


**Figure S12-1:** TEM images of uranyl acetate-stained nanoparticles self-assembled from a solution of $PMA_{231}$-*b*-PSPA-$Na_{48}$ ($x_{SPA\text{-}Na}$ = 0.17) at 1 g $L^{-1}$ in 10 mM $KNO_3$.

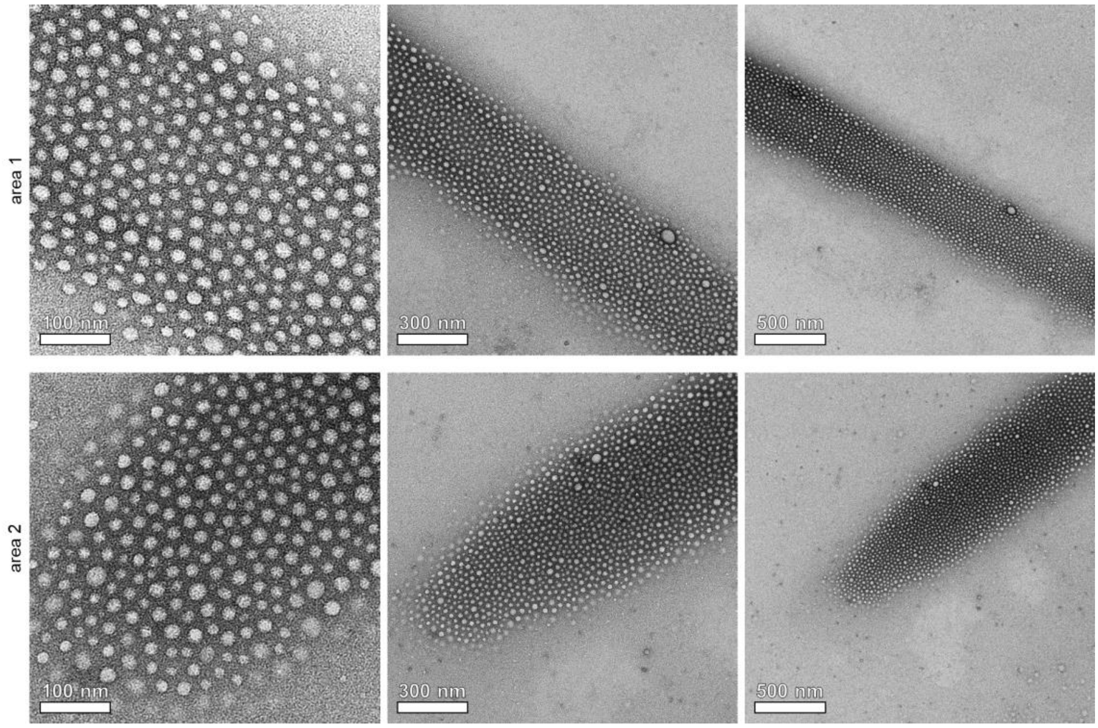


**Figure S12-2:** TEM images of uranyl acetate-stained nanoparticles self-assembled from a solution of $PMA_{193}$-*b*-PSPA-$Na_{94}$ ($x_{SPA\text{-}Na}$ = 0.33) at 1 g $L^{-1}$ in 10 mM $KNO_3$.

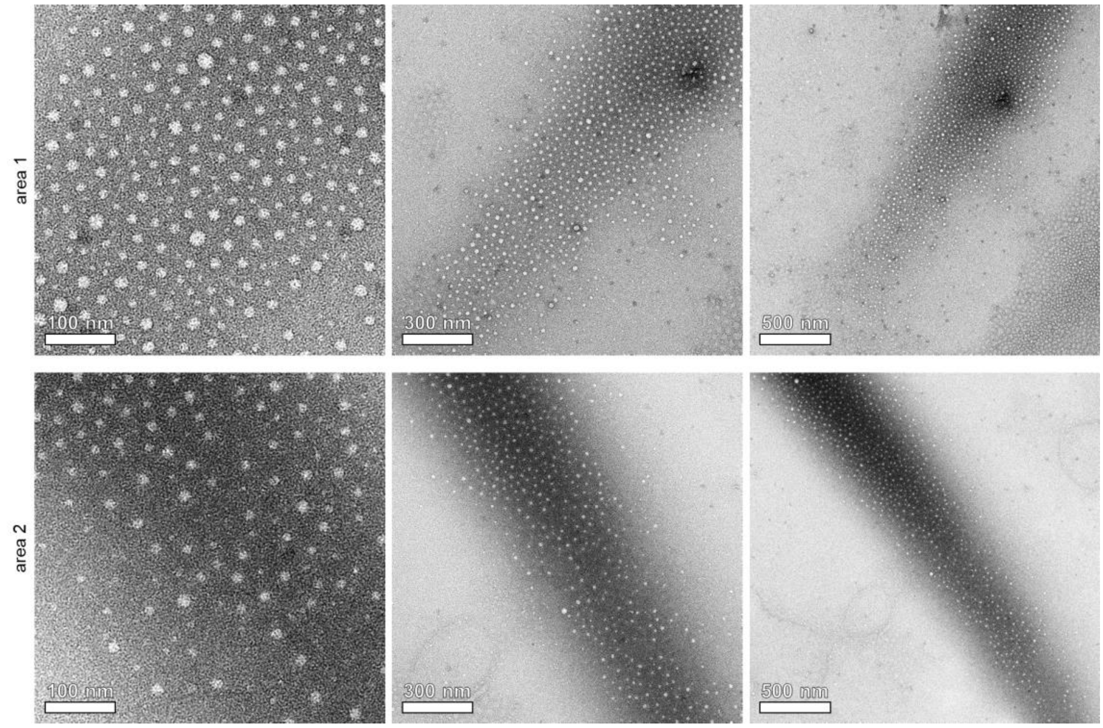


**Figure S12-3:** TEM images of uranyl acetate-stained nanoparticles self-assembled from a solution of $PMA_{146}$-*b*-PSPA-$Na_{164}$ ($x_{SPA\text{-}Na}$ = 0.53) at 1 g $L^{-1}$ in 10 mM $KNO_3$.

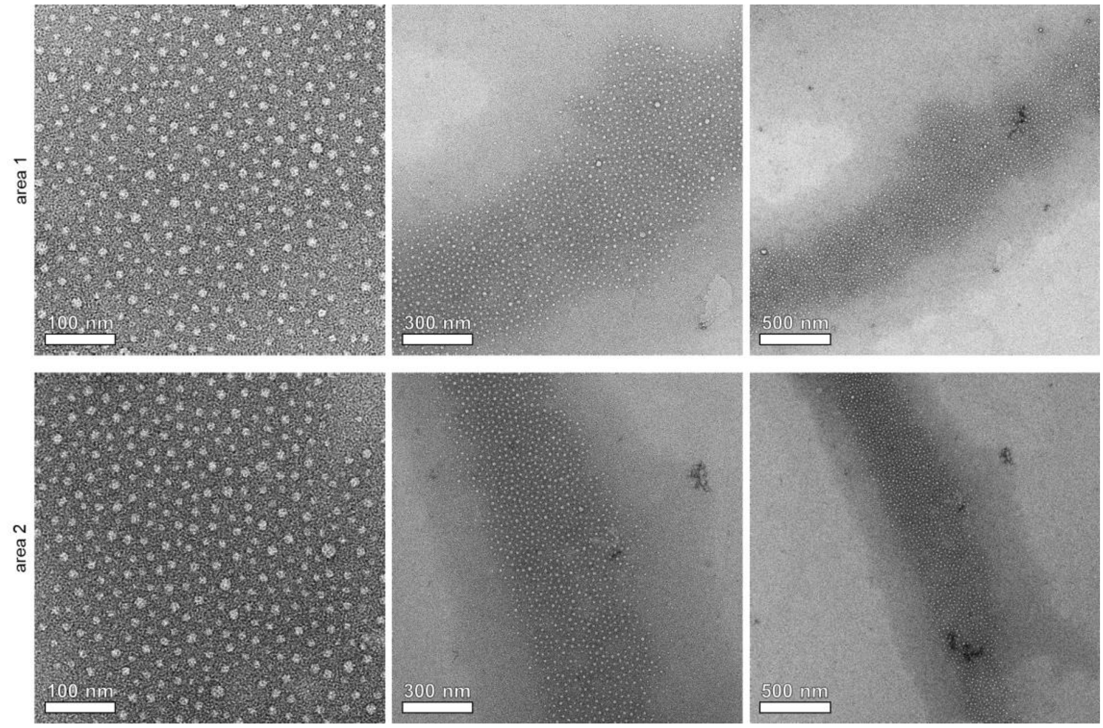


**Figure S12-4:** TEM images of uranyl acetate-stained nanoparticles self-assembled from a solution of $PMA_{98}$-*b*-PSPA-$Na_{228}$ ($x_{SPA\text{-}Na}$ = 0.70) at 1 g $L^{-1}$ in 10 mM $KNO_3$.

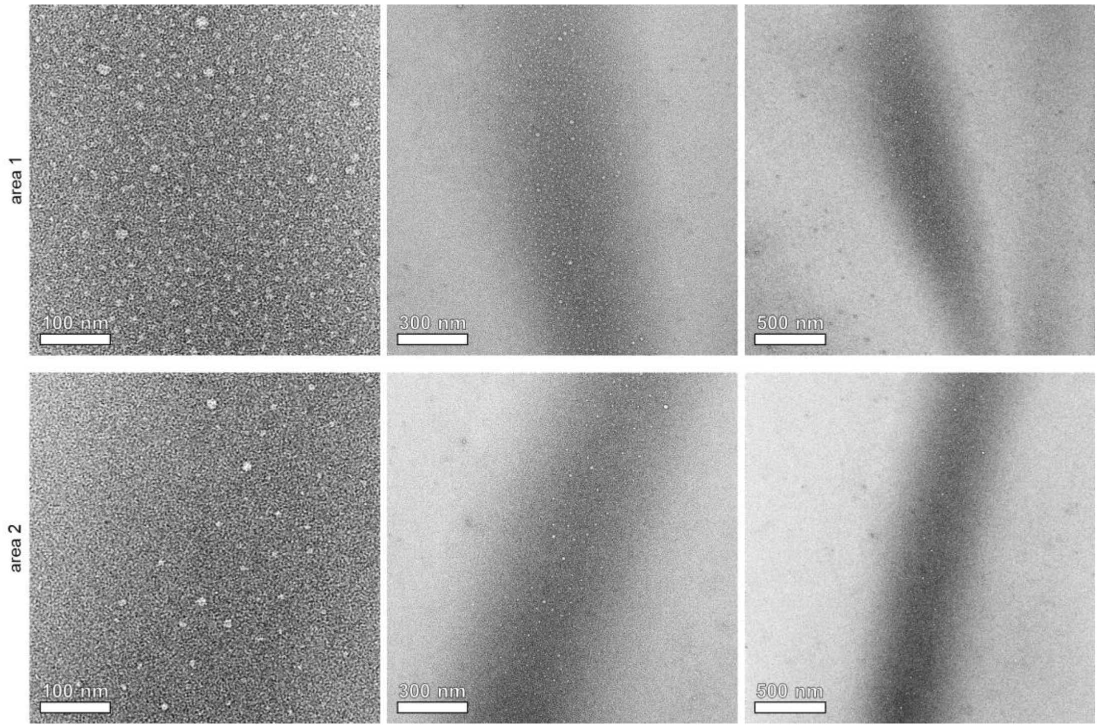


**Figure S12-5:** TEM images of uranyl acetate-stained nanoparticles self-assembled from a solution of $PMA_{47}$-*b*-PSPA-$Na_{254}$ ($x_{SPA\text{-}Na}$ = 0.84) at 1 g $L^{-1}$ in 10 mM $KNO_3$.

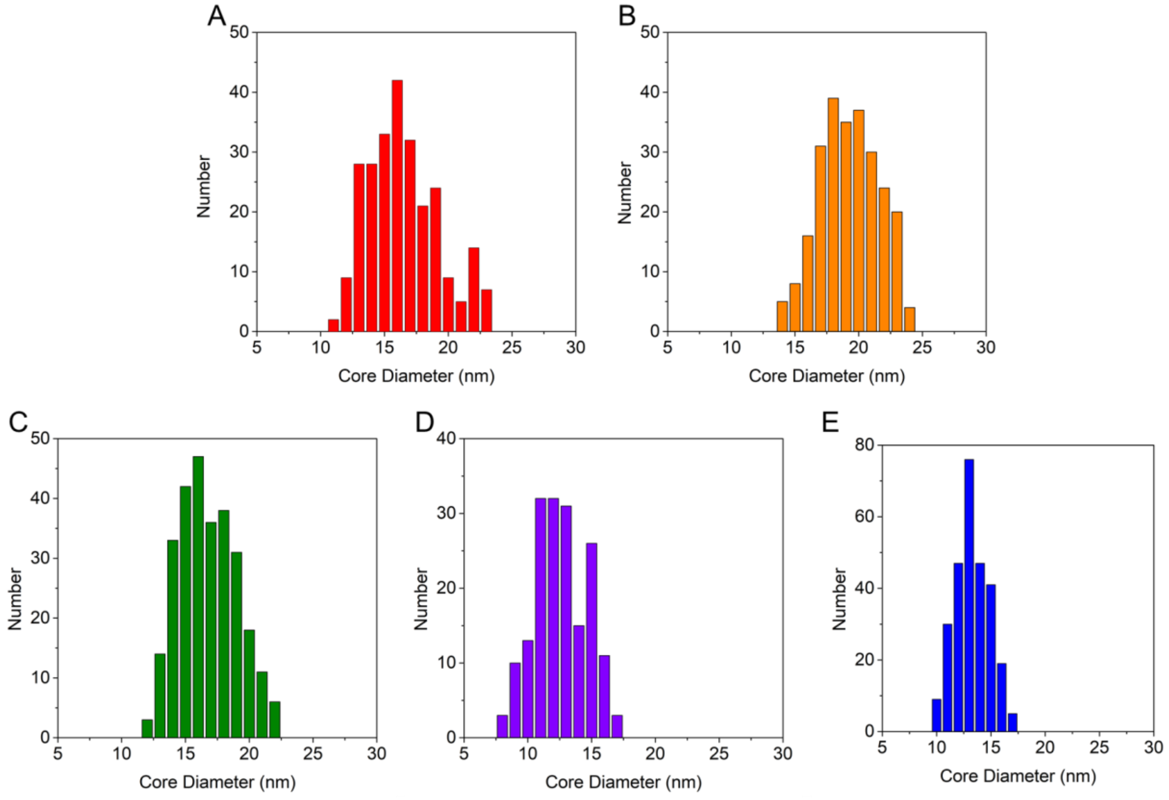

**Figure S12-6:** Statistical analyses of the core diameter of nanoparticles achieved from the self-assembly of (A) $PMA_{231}$-*b*-PSPA-$Na_{48}$, (B) $PMA_{193}$-*b*-PSPA-$Na_{94}$, (C) $PMA_{146}$-*b*-PSPA-$Na_{164}$, (D) $PMA_{98}$-*b*-PSPA-$Na_{228}$ and (E) $PMA_{47}$-*b*-PSPA-$Na_{254}$ in 10 mM $KNO_3$. Data extracted from 150 to 250 specimens across multiple TEM images.

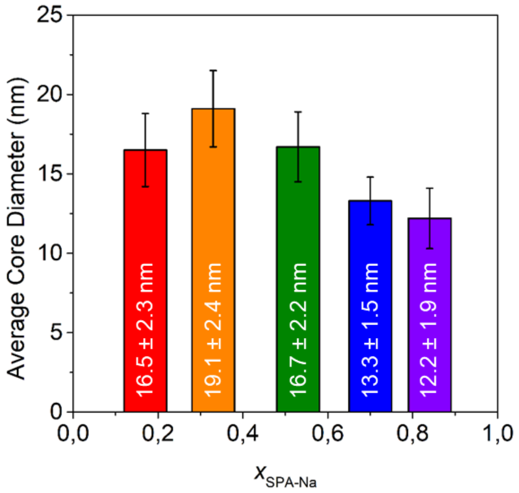


**Figure S12-7:** Average core diameter of the self-assembled nanoparticles achieved from $PMA_{231}$-*b*-PSPA-$Na_{48}$ (red), $PMA_{193}$-*b*-PSPA-$Na_{94}$ (orange), $PMA_{146}$-*b*-PSPA-$Na_{164}$ (green), $PMA_{98}$-*b*-PSPA-$Na_{228}$ (blue) and $PMA_{47}$-*b*-PSPA-$Na_{254}$ (violet) in 10 mM $KNO_3$ as a function of the $x_{SPA\text{-}Na}$ molar fraction. Data extracted from 150 to 250 specimens across multiple TEM images.

**S13: SAXS of the polymer micelles in aqueous solution.**

The fitted curves are reported in Figure S13 below and the fitted values for the core-shell particles are summarized in Table S11. The core size is in good agreement with the TEM analysis and the shell thickness scales according to the degree of polymerization of the SPA block. The scattering length density of the core was first derived from the fitting of the $PMA_{193}$-*b*-PSPA-$Na_{94}$ sample ($x_{SPA-Na}$ = 0.33, sample showing the most well-defined SAXS profile) and was kept constant during fitting for the other samples. The SLD for the solvent at room temperature was used as $\eta_m = 0.94 \times 10^{11}$ $cm^{-2}$. The contrast of between the core and the solvent was calculated as $\eta_{core}-\eta_m = 0.16 \times 10^{11}$ $cm^{-2}$ (based on the composition of the PMA) and kept constant during the fitting procedure. Conversely the shell scattering length density is much lower than the one expected for bulk SPA and is much closer to the solvent (*i.e.,* 10 mM $KNO_3$, ≈ water) value, meaning that the shell is highly swollen, as expected for charged systems. The background exponent $\alpha$ was found to oscillate between 1.3 and 2.3. For $PMA_{47}$-*b*-PSPA-$Na_{254}$, the total particle size seems to be outside of the measured range and only scattering from the outer PSPA-Na corona is measured.

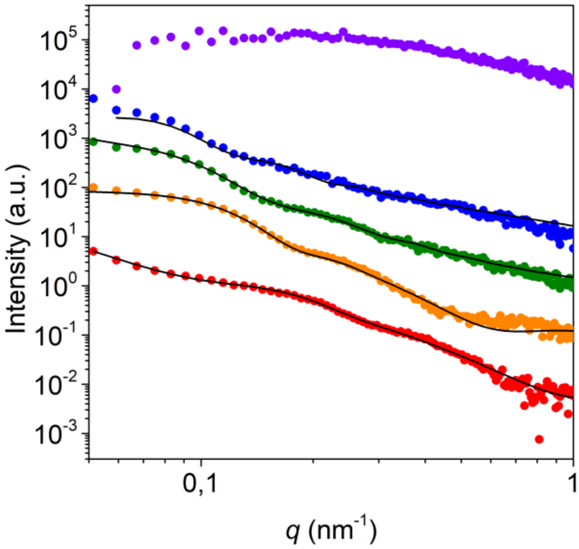


**Figure S13:** Comparative SAXS profiles of the nanoparticles produced through solution self-assembly of $PMA_{231}$-*b*-PSPA-$Na_{48}$ ($x_{SPA-Na}$ = 0.17, red), $PMA_{193}$-*b*-PSPA-$Na_{94}$ ($x_{SPA-Na}$ = 0.33, orange), $PMA_{146}$-*b*-PSPA-$Na_{164}$ ($x_{SPA-Na}$ = 0.53, green), $PMA_{98}$-*b*-PSPA-$Na_{228}$ ($x_{SPA-Na}$ = 0.70, blue) and $PMA_{47}$-*b*-PSPA-$Na_{254}$ ($x_{SPA-Na}$ = 0.84, violet) in 10 mM $KNO_3$. Mathematical fits (black lines) are depicted when available.

**Table S11:** Summary of the structural parameters obtained by fitting the SAXS data.

| $x_{SPA\text{-}Na}$ (mol. %) | $\eta_{SPA\text{-}Na}$-$\eta_m$ ($10^{11}$ cm$^{-2}$) | $R_{core}$ (nm) | $R_{shell}$ (nm) | $\sigma_c$ (%) | background |
|---|---|---|---|---|---|
| 17 | -0.170 | 16.7 | 6.0 | 16 | $3.50*10^{-3}+1*10^{-3}*q^{-2.66}$ |
| 33 | -0.070 | 24.6 | 9.1 | 21 | 0.11 |
| 53 | -0.017 | 16.9 | 40.7 | 50 | $0.82+0.65*q^{-2.21}$ |
| 70 | -0.006 | 12.7 | 57.6 | 50 | $1.00+15.44*q^{-1.26}$ |

The core size polydispersity was described by a Schultz-Zimm distribution function. $\eta_{SPA\text{-}Na}$-$\eta_m$: scattering length difference between the SPA-Na phase and the surrounding media. $R_{core}$: core radius. $R_{shell}$: outer shell thickness. $\sigma_c$ : core polydispersity. background: Background function.

**S14: Schematic representation of the 'sampling' method.**

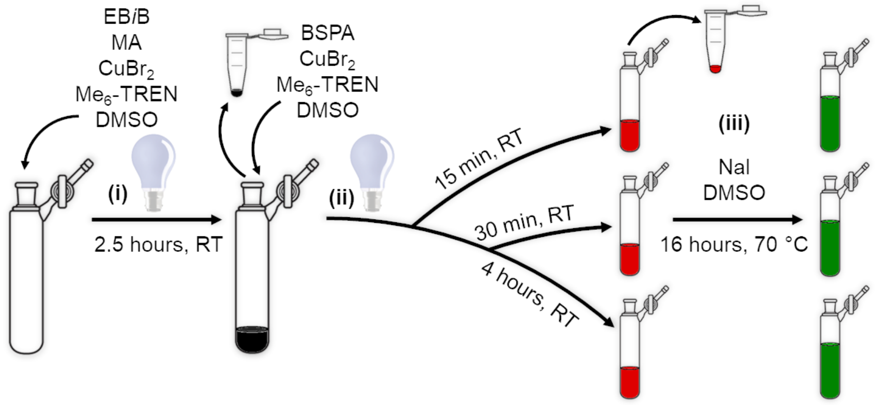


**Figure S14:** Schematic representation of the synthesis of several amphiphilic block copolymers using a 'sampling' method, including (i) the production of the first PMA block, followed by (ii) chain extension with BSPA monomer and (iii) deprotection of each block copolymer after sampling.

**S15: $^1$H NMR of the block copolymers achieved through sampling.**

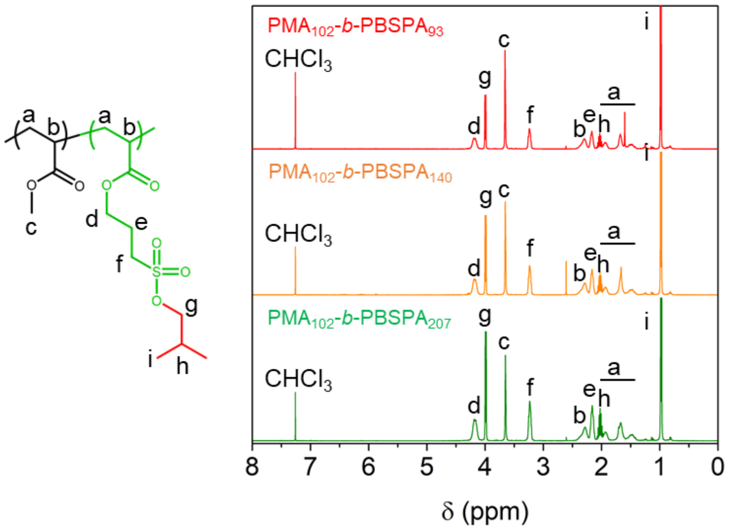


**Figure S15-1:** $^1$H NMR spectra of the three protected block copolymers achieved in one-pot polymerization through sampling.

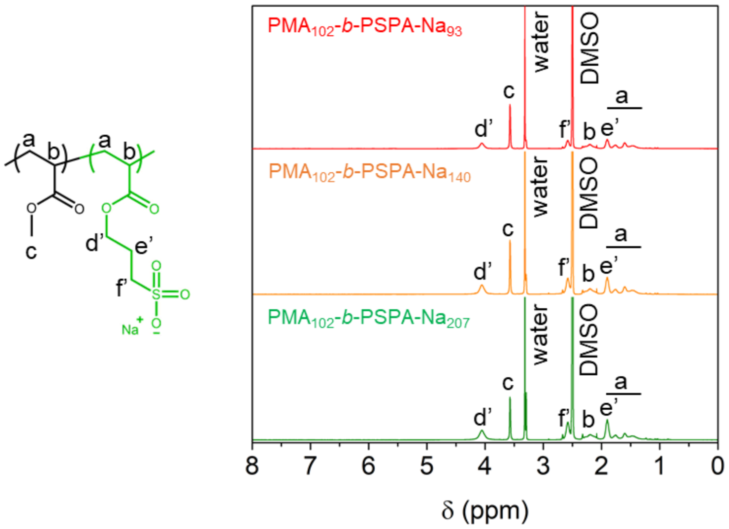


**Figure S15-2:** $^1$H NMR spectra of the three deprotected amphiphilic block copolymers achieved in one-pot polymerization through sampling.

**S16: DLS analyses of the amphiphilic BCPs produced through sampling.**

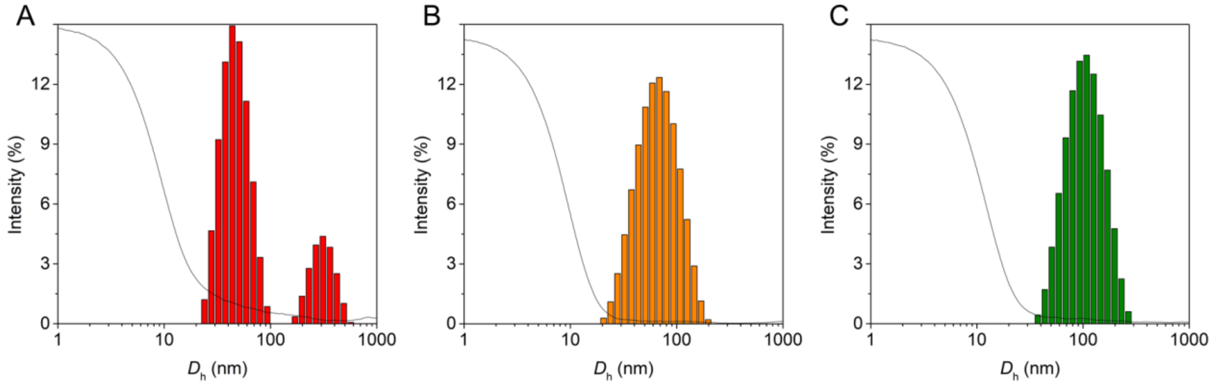


**Figure S16:** Dynamic light scattering intensity plots (bars) and corresponding correlogram functions (solid lines) of (A) $PMA_{103}$-*b*-PSPA-$Na_{93}$, (B) $PMA_{103}$-*b*-PSPA-$Na_{140}$ and $PMA_{103}$-*b*-PSPA-$Na_{207}$ nanoparticles solutions at 1 g $L^{-1}$ in $KNO_3$.

## S17: Electron microscopy images of the amphiphilic BCPs produced through sampling.

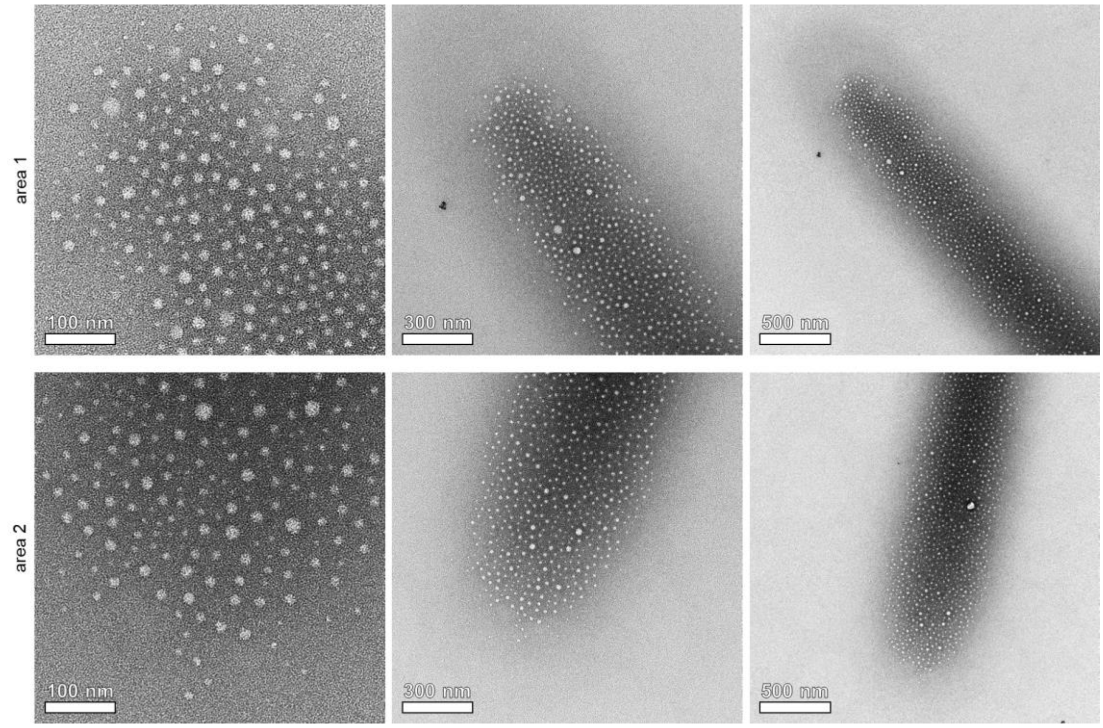


**Figure S17-1:** TEM images of uranyl acetate-stained nanoparticles self-assembled from a solution of $PMA_{103}$-*b*-$PSPA\text{-}Na_{93}$ ($x_{SPA\text{-}Na}$ = 0.48) at 1 g $L^{-1}$ in 10 mM $KNO_3$.

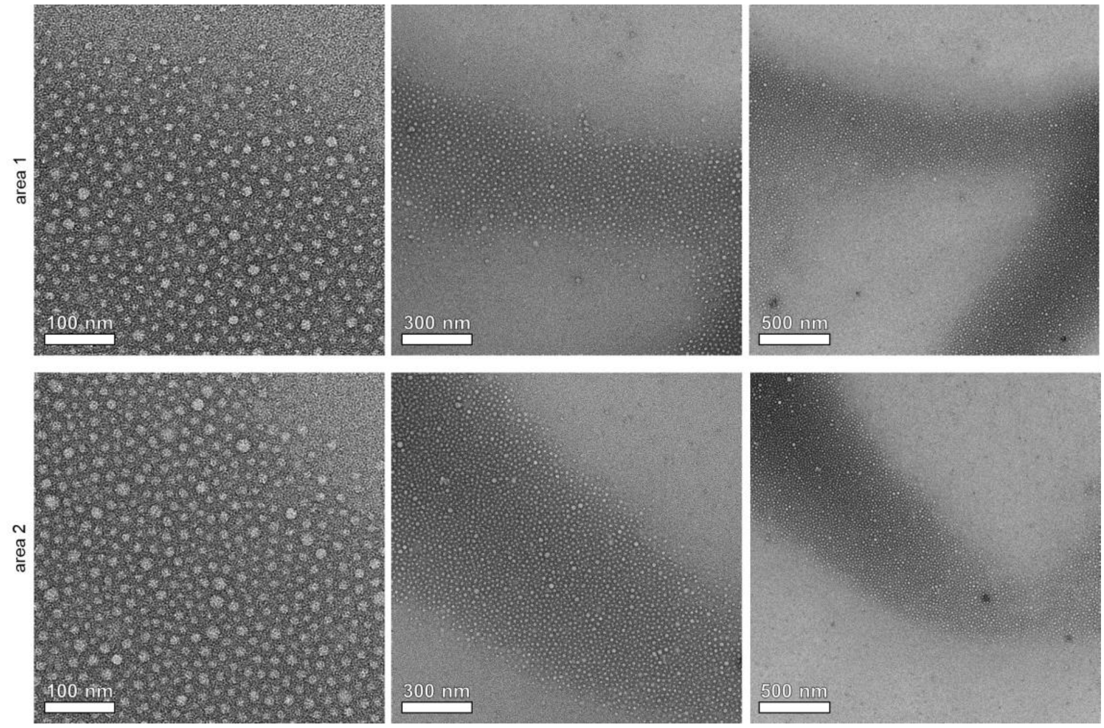


**Figure S17-2:** TEM images of uranyl acetate-stained nanoparticles self-assembled from a solution of $PMA_{103}$-*b*-$PSPA\text{-}Na_{140}$ ($x_{SPA\text{-}Na}$ = 0.58) at 1 g $L^{-1}$ in 10 mM $KNO_3$.

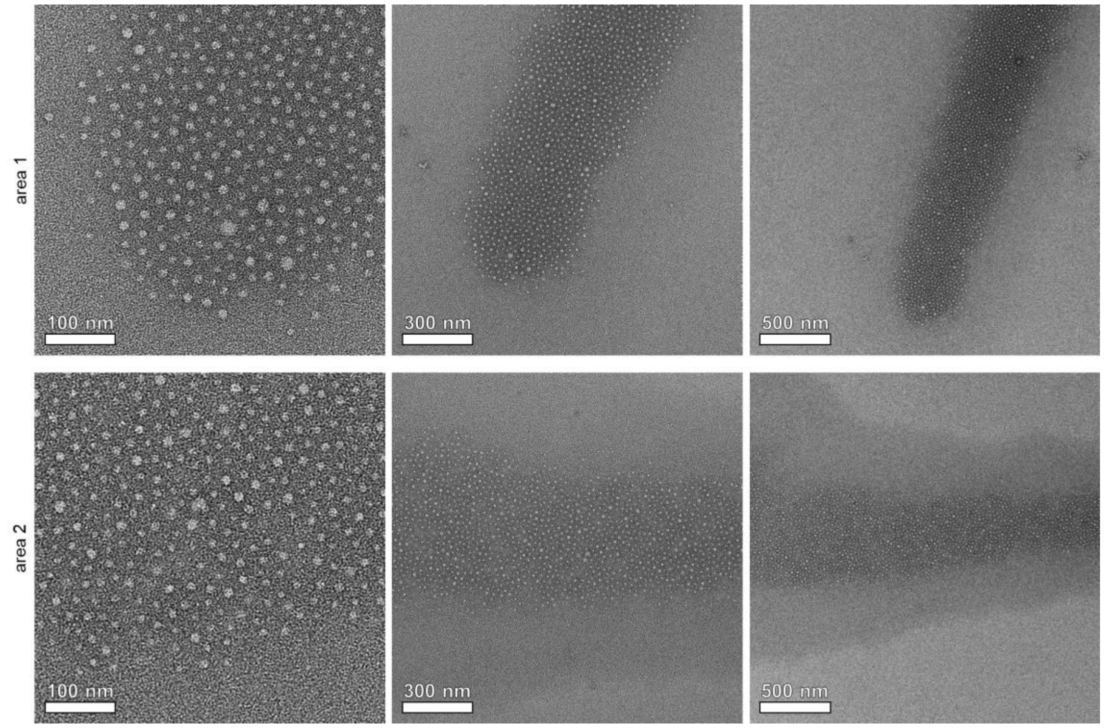


**Figure S17-3:** TEM images of uranyl acetate-stained nanoparticles self-assembled from a solution of PMA$_{103}$-*b*-PSPA-Na$_{207}$ ($x_{SPA\text{-}Na}$ = 0.67) at 1 g L$^{-1}$ in 10 mM KNO$_3$.

## Supporting References

[1] *Macromolecules* **2022**, 55, 19, 8795–8807

[2] *J. Appl. Cryst*. **2015**, 48, 1587–1598
